# Geometric alignment of aminoacyl-tRNA relative to catalytic centers of the ribosome underpins accurate mRNA decoding

**DOI:** 10.1038/s41467-023-40404-9

**Published:** 2023-09-11

**Authors:** Dylan Girodat, Hans-Joachim Wieden, Scott C. Blanchard, Karissa Y. Sanbonmatsu

**Affiliations:** 1 Theoretical Biology and Biophysics, Theoretical Division, Los Alamos National Laboratory, Los Alamos, NM 87545 USA; 2 Department of Microbiology, University of Manitoba, Winnipeg, MB R3T 2N2 Canada; 3 Department of Structural Biology, St. Jude Children’s Research Hospital, Memphis, TN 38105 USA; 4 New Mexico Consortium, Los Alamos, NM 87545 USA; 5 Present Address: Department of Chemistry and Biochemistry, University of Arkansas, Fayetteville, AR 72701 USA

**Keywords:** Computational biophysics, Biological physics, Cryoelectron microscopy

## Abstract

Accurate protein synthesis is determined by the two-subunit ribosome’s capacity to selectively incorporate cognate aminoacyl-tRNA for each mRNA codon. The molecular basis of tRNA selection accuracy, and how fidelity can be affected by antibiotics, remains incompletely understood. Using molecular simulations, we find that cognate and near-cognate tRNAs delivered to the ribosome by Elongation Factor Tu (EF-Tu) can follow divergent pathways of motion into the ribosome during both initial selection and proofreading. Consequently, cognate aa-tRNAs follow pathways aligned with the catalytic GTPase and peptidyltransferase centers of the large subunit, while near-cognate aa-tRNAs follow pathways that are misaligned. These findings suggest that differences in mRNA codon-tRNA anticodon interactions within the small subunit decoding center, where codon-anticodon interactions occur, are geometrically amplified over distance, as a result of this site’s physical separation from the large ribosomal subunit catalytic centers. These insights posit that the physical size of both tRNA and ribosome are key determinants of the tRNA selection fidelity mechanism.

## Abstract (web summary)

Protein synthesis is dependent on the ribosome’s ability to accurately select tRNA. Molecular simulations reveal divergent pathways for correct and incorrect tRNA during selection, indicating that tRNA alignment is key to protein production.

## Introduction

Faithful translation of genetic information in the form of messenger RNA (mRNA) into proteins is determined by the ability of ribosomes to select for cognate aminoacyl(aa)-tRNA for each mRNA codon from the much larger pool of those that are near-cognate (one mismatch) or non-cognate (more than one mismatch). Errors in this process, occur at a frequency of 10^−2^ to 10^−6^, depending on species and varying on codon-anticodon mismatch identity^1–7^. result in erroneous peptides, leading to aggregation products, cell death, or neurodegenerative diseases^8,9^. The essentiality of faithful tRNA selection to cellular viability is highlighted by the functions of clinically important antibiotics that specifically impair the ribosome’s selectivity^10–12^. Such considerations have fueled extensive efforts to gain a deeper, fundamental understanding of how ribosomes achieve fidelity to thereby establish the genetic code.

A kinetic proofreading mechanism of aa-tRNA selection in prokaryotic organisms^13–16^ has been validated using a battery of ensemble biochemical investigations spanning multiple decades^17–28^ including, but not limited to, X-ray crystallography^29–51^, rapid kinetics, single-molecule fluorescence resonance energy transfer (smFRET) measurements and cryogenic electron microscopy (cryo-EM) imaging^52–79^. These efforts have shown that the initial selection phase of aa-tRNA selection commences when the ternary complex of Elongation Factor (EF)-Tu, guanosine-5’-triphosphate (GTP), and aa-tRNA binds to the aminoacyl (A) site of the ribosome, in the absence of codon-anticodon interactions and prior to EF-Tu docking with the large subunit (LSU) GTPase activating center (GAC) (an initial binding (IB) or pre-A/T state)^76–80^. The pre-A/T nomenclature refers to the approach of aa-tRNA to the small subunit (SSU) A site of the ribosome while remaining bound to EF-Tu in the ternary complex. Subsequently, the aa-tRNA anticodon samples configurations until it forms potential base pairs with the mRNA codon. The formation of codon-anticodon base pairs (a codon-recognition (CR) state) precipitate compacted conformations of the SSU of the ribosome^29^, where the universally conserved A1492, A1493, and G530 bases of the SSU 16S rRNA form interactions with the mini-helix formed by mRNA-codon-tRNA anticodon base pairs^28–31,79,81–85^. These critical initial selection events, together with aa-tRNA “bending”^59–62,79^, allow EF-Tu(GTP) to productively dock against the Sarcin-Ricin loop (SRL) within the GAC of the LSU, (a GTPase activated (GA) or A/T state) in which GTP hydrolysis occurs^76,77^. Near- and non-cognate aa-tRNA are less efficient at GTPase activation and preferentially dissociate from the ribosome prior to energy consumption due to their reduced codon-anticodon base pair lifetimes^76,77,86,87^.

Proofreading selection, after EF-Tu-catalyzed GTP hydrolysis, begins with the liberation of inorganic, γ-phosphate (P_i_) from EF-Tu and is fueled by torsional energy within a ‘bent’ aa-tRNA conformation^88^. P_i_ release and coupled conformational changes in EF-Tu(GDP), including its switch I element, allow the 3’-CCA end of aa-tRNA to navigate towards the LSU A site and the peptidyl transferase center (PTC) via the accommodation corridor^66,88–91^. Separation of the 3’-CCA end of aa-tRNA from EF-Tu(GDP) enables the complete release of EF-Tu(GDP) from aa-tRNA and the ribosome to either allow accommodation of the 3’-CCA end into the PTC of the LSU or aa-tRNA dissociation prior to peptide bond formation. Successful docking of aa-tRNA within the PTC culminates in the transfer of the nascent polypeptide chain to the 3’-CCA end of the incoming tRNA, producing a pre-translocation complex, in which peptidyl-tRNA is “classically” (A/A) positioned. Cognate and near-cognate aa-tRNAs can, with low efficiencies, successfully navigate the initial selection and proofreading mechanisms to achieve similar “endpoint” positions^92^. Delineating how cognate and near-cognate aa-tRNA differentially traverse initial selection and proofreading is paramount to establishing physical insights into how fidelity is achieved during protein synthesis and how antibiotics affect these processes.

Here, we simulated cognate and near-cognate aa-tRNA selection on the *E. coli* ribosome using all-atom structure-based models to investigate the dynamic repositioning of aa-tRNA during initial selection and proofreading. Prior simulations have successfully examined cognate aa-tRNA dynamics during the accommodation step of proofreading selection^91,93–101^. The present investigations examine initial selection and proofreading selection processes for both cognate or near-cognate aa-tRNA bound to EF-Tu, before and after GTP hydrolysis, respectively. In this context, we find that both cognate and near-cognate aa-tRNA enter the catalytic centers of the LSU via pivot-like motions of the incoming tRNA elbow region and acceptor stem relative to the codon-anticodon pair within the SSU decoding region. Steric distinctions in the codon-anticodon pair result in near-cognate aa-tRNA exhibiting a higher likelihood of adopting misaligned positions with respect to the GAC, SRL, accommodation corridor, and PTC compared to cognate aa-tRNA. These distinctions are observed during initial selection and during proofreading. We find that the misalignment signatures of near-cognate aa-tRNA are suppressed in the presence of aminoglycosides that promote miscoding.

These findings are globally consistent with smFRET imaging of tRNA selection^77^ and a physical framework in which the angular distribution of pivot angles sampled by near-cognate aa-tRNA is more divergent than those of cognate aa-tRNA. In this model, the narrow cognate aa-tRNA angular distribution gives rise to amplified probabilities of achieving positions that are appropriately aligned with the centers responsible for GTP hydrolysis and peptide bonding formation. These insights suggest that both the sizes and the flexibilities of the aa-tRNA molecule and the ribosome are critical determinants of the fidelity mechanism and that these considerations contribute to the conservation of these parameters throughout evolution.

## Results

### Simulations of tRNA initial selection and proofreading

Investigations of tRNA initial selection and proofreading structural dynamics require adequate selection of an accurate starting model for molecular simulations. SmFRET, X-ray crystallography, and cryo-EM investigations of aa-tRNA selection have revealed distinct intermediates states of the EF-Tu(GTP) aa-tRNA ternary complex binding during initial selection^66,73,76–79^. Initial structural studies resolved ternary complex bound to the ribosome in a GA state, to reveal endpoint codon-anticodon interactions within the SSU decoding center, the nature of aa-tRNA bending, and the contact points between EF-Tu and the LSU involved in GTP hydrolysis^36,37,52,59–62,68,70^. More recently, codon-anticodon interactions have been defined by cryo-EM corresponding to IB, CR, and GA states, as defined by earlier smFRET studies^78,79^.

**Fig. 1:**
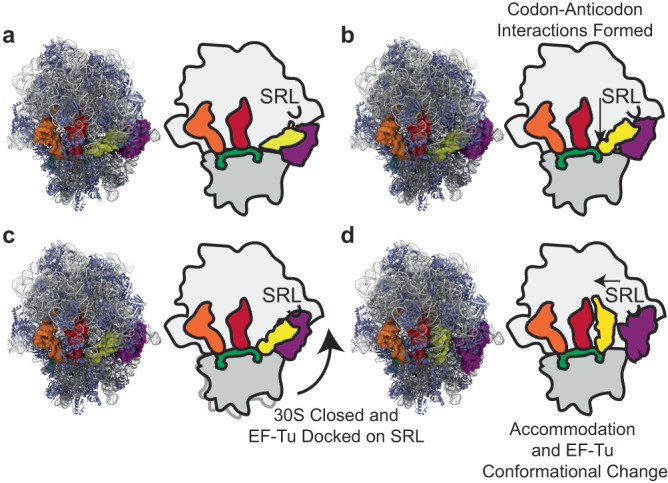
The conformations of 70S•EF-Tu•aa-tRNA complex. **a** Structures of the cognate ternary complex bound to 70S ribosome (left) and corresponding schematic diagrams for cognate and near-cognate (right). Grey, rRNA; light blue, ribosomal proteins; yellow, A-site tRNA; red, P-site tRNA; orange, E-site tRNA; green, mRNA. (A) The IB state (pre-A/T aa-tRNA position or I or C_2_): codon-anticodon interactions not formed, and EF-Tu not engaged with the SRL. **b** CR state: codon-anticodon interactions formed and EF-Tu not engaged with SRL (A/T aa-tRNA position or II or C_3_). **c** GA state: codon-anticodon interactions formed and EF-Tu engaged with SRL (A/T aa-tRNA position III or C_4_). **d** AC state: tRNA accommodated into the A/A position and EF-Tu has undergone a conformational change (A/A aa-tRNA position). In brackets are the labeling schemes from Loveland et al. 2017 and Fislage et al. 2018, respectively^78,79^.

The recent IB, CR, and GA structures were used as initial, native-state models in our structure-based simulations, with cognate Phe-tRNA^Phe^ and near-cognate Lys-tRNA^Lys^ (Fig. 1a–c)^79^. In the near-cognate Lys-tRNA^Lys^ simulations, codon-anticodon interactions were modeled to contain a G-U mismatch at the middle base-pair. The G-U mismatch can form a base-pair that mimics Watson Crick interactions to induce miscoding at a higher frequency^1,7,21,32–34,102^. A fully accommodated (AC) position of the aa-tRNA in a pre-translocation complex was set as the A/A state with the same coordinates for cognate and near-cognate aa-tRNA (Fig. 1d). Due to the limitations of structure-based simulation approaches that do not include quantum mechanical considerations^103^, we did not simulate GTP hydrolysis. Rather, we simulated tRNA selection from IB to AC states with EF-Tu loaded with GDP in an active, GTP-bound conformation, while freely allowing EF-Tu to undergo conformational changes (Methods). As simulations were initiated in a GTP-bound conformation and allowed to access rearrangements in EF-Tu that are accelerated by GTP hydrolysis, GTP hydrolysis is not simulated due to challenges presently associated with integrating quantum mechanical calculations.

**Fig. 2:**
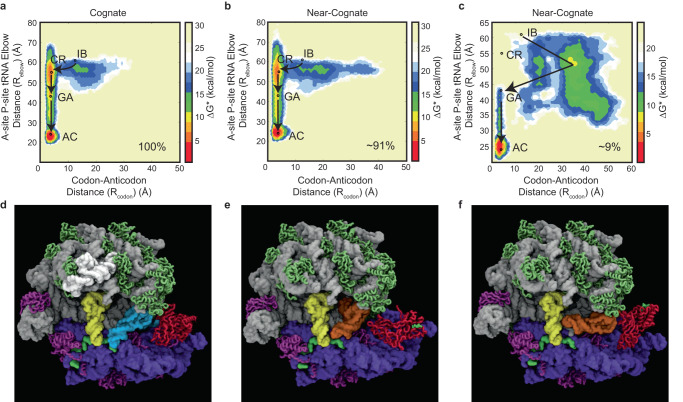
Near-cognate aa-tRNA exhibits alternative pathways of motion during initial selection. Approximate free-energy landscapes of aa-tRNA accommodation along the reaction coordinate R_elbow_ and R_codon_. **a** Cognate and (**b**) near-cognate tRNA accommodation from structure based-simulations starting from the IB state and following a path that passes through CR, GA, to the AC state. **c** Near-cognate aa-tRNA accommodation following an alternative, elbow-first path proceeding through the IB, GA, and AC states, bypassing codon recognition (9% of simulations). **d** Image of cognate aa-tRNA (blue) bound to EF-Tu (red) is delivered to the SSU of the ribosome (blue and purple), codon-anticodon base-pairs with the mRNA (green), and the aa-tRNA form prior to the GA state. **e** Image of near-cognate aa-tRNA (orange) selection where codon-anticodon base-pairs with the mRNA form at the GA state. **f** Image of near-cognate aa-tRNA (orange) non-productive selection where codon-anticodon base-pairs do not appear to form and aa-tRNA fails to accommodate.

Approximate free energy landscapes were employed to measure the progression of cognate aa-tRNA through the tRNA selection pathway (IB-CR-GA-AC). These landscapes were compiled from the reaction coordinates, R_elbow_ and R_codon_ (Fig. 2a–c). R_elbow_ describes the distance between the A-site and P-site tRNA elbow, a measure of aa-tRNA progression into the ribosome; R_codon_ describes the distance between the mRNA codon and the tRNA anticodon (Methods). Free-energy landscapes can be approximated from Boltzmann weighted probability distributions. With current MD strategies, however, it is extremely difficult to ascertain if sufficient sampling has occurred, even at convergence. For example, dynamics observed on the ns to ms timescale could explore additional conformations if sampling was extended beyond these timescales. Therefore, the landscapes we describe represent approximate free energy landscapes.

Cognate aa-tRNA simulations began in an IB configuration (pre-A/T aa-tRNA), where ternary complex was bound through contacts with an open SSU (separated shoulder and body domains), while EF-Tu interactions with the SRL and mRNA codon-tRNA anticodon base-pairing were both absent (Fig. 1a). Ternary complex positions were dynamic within the IB state and the mRNA codon and tRNA anticodon separation distance was broadly distributed (Fig. 2a). From the IB state, the codon-anticodon separation distance collapsed, prior to EF-Tu establishing stable interactions with the LSU GAC and SRL (Fig. 2a), consistent with CR state formation (Fig. 1b). SSU shoulder and body domain closure subsequently moved the aa-tRNA elbow into the ribosome and towards the P site, forming the GA state (Fig. 1c), in which EF-Tu engaged the SRL and aa-tRNA was torsionally restrained in a ‘bent’ (A/T) position (Fig. 2a). Consistent with pathways similar to those originally described by smFRET^76,77^, cognate aa-tRNA exhibited spontaneous, stochastic aa-tRNA transitions from the IB to AC states (Fig. 1a–d), passing through CR and GA states in sequential order (Fig. 2a, d).

To assess the convergence of the simulations, we calculated a pointwise RMSD between successive energy landscapes as a function of greater and greater sampling (${Conv}\left(t\right)$ or $\zeta \left(t\right)$) to demonstrate that the simulations converged after approximately 100 million-time steps (Supplementary Figs. 1, 2). The simulations are considered to approach convergence when ${Conv}\left(t\right)$ approaches a plateau or $\zeta \left(t\right)$ approaches zero^81^. Simulations of up to 250 million-time steps did not display a change in the plateau of ${Conv}\left(t\right)$ or $\zeta \left(t\right)$ value and thus timesteps beyond 100 million-time steps were not included in the analysis. Using similar strategies to determine replica exchange simulations timescales^104^, the average diffusion time, of the tRNA elbow (diffusion coefficient = ~0.1Å^2^/t_ru_) through the tRNA accommodation corridor was estimated to be τ_ru_ ~ 1 ns which reflects biochemically determined rates of aa-tRNA accommodation and are consistent with prior structure-based simulations (Supplementary Material)^96,97,105^.

### Near-cognate aa-tRNA exhibits alternative pathways of motion during initial selection

Comparative analyses revealed that both cognate and near-cognate aa-tRNA simulations typically followed globally similar pathways (91% of simulations) (Fig. 2a, b; Supplementary Movie 1). However, near-cognate substrates adopt a broader range of codon-anticodon and inter-elbow distance separations within both the IB and CR states (Fig. 2b). These data are indicative of both a codon dependency on the formation of the CR state as well as codon-anticodon dependent impacts on how aa-tRNA moves into the ribosome during the selection process. Entirely alternative pathways of motion into the ribosome were also evidenced (9% of simulations) that correlated with a broader range of separation distances between the mRNA codon and tRNA anticodon pair while elbow distance separation decreased (Fig. 2c, e, f; Supplementary Movies 2, 3). These globally alternative near-cognate aa-tRNA pathways entered the ribosome via a broad array of ‘elbow-first’ orientations in the absence of any codon-anticodon base-pairing, or were non-productive in aa-tRNA selection, where codon-anticodon base-pairs fail to form. The increased divergence of near-cognate aa-tRNA motion is qualitatively consistent with smFRET imaging investigations of aa-tRNA selection, which revealed a broader distribution of near-cognate aa-tRNA positions during tRNA selection than that observed for cognate aa-tRNA^77^. As the simulations reveal, near-cognate aa-tRNA can adopt rare and likely energetically unstable positions during selection that arise downstream of locally misaligned positions in which codon-anticodon interactions are either not formed or mis-formed (Fig. 2f). Due to the high energy state (lack of defined interactions between the tRNA and ribosome and large repulsive force of tRNA rRNA negative charges) of the tRNA to adopt an unconventional position in the simulations (Fig. 2f) these tRNA are expected to either inefficiently complete or ultimately fail to complete, the selection process as observed by smFRET^77^. Moreover, as aa-tRNA misalignment commences during the initial selection, these observations also help explain why near-cognate selection events exhibit reduced rates of GTP hydrolysis and peptide bond formation^86,87^.

To examine the dependency of these alternative pathways on codon-anticodon pairing in a context relevant to the fidelity mechanism, we performed simulations in the presence of the aminoglycoside gentamycin (GEN) and neomycin (NEO) that promote miscoding by repositioning residues A1492 and A1493 within the h44 decoding center^106–110^. Simulations of near-cognate accommodation performed with GEN or NEO, engaged with h44 of the decoding center proximal to the A1492 and A1493 monitoring bases, abolished the observed alternative “elbow domain”-first events (Supplementary Fig. 3). These findings suggest that both 4,5- and 4,6-linked aminoglycoside class antibiotics may help ensure the formation of more native-like codon-anticodon interactions between the mRNA and aa-tRNA before ternary complex begins its trajectory of motion into the ribosome. They are also consistent with monitoring base interactions (16S rRNA G530, A1492, and A1493), with the codon-anticodon base-pairs being central to aa-tRNA selection events beyond domain closure^30^.

### Near-cognate aa-tRNA exhibits misalignment prior to its release from EF-Tu

**Fig. 3:**
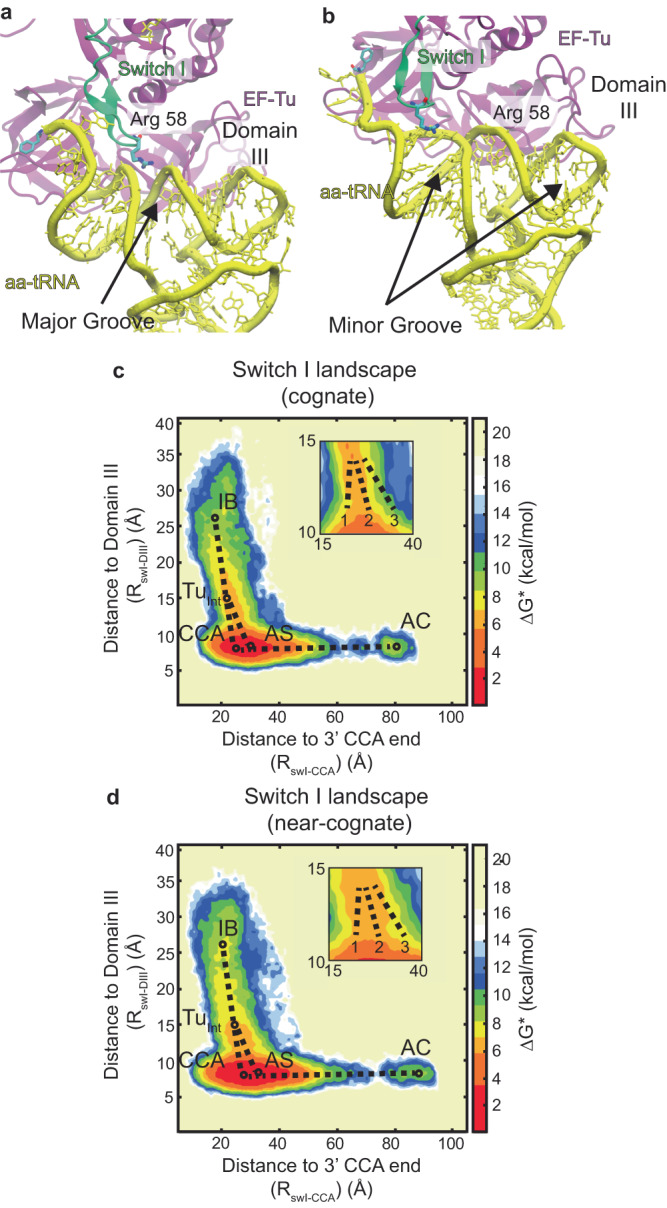
Near-cognate aa-tRNA displays a larger distribution of positioning during release from EF-Tu. **a** Pathway of switch I of EF-Tu through the major groove of the cognate acceptor stem tRNA helix (AS). **b** Pathway of switch I of EF-Tu through the minor groove of the near-cognate acceptor stems near the 3’ CCA end or elbow of the tRNA. Approximate free energy landscape of switch I traversing the accommodating tRNA for (**c**) cognate and (**d**) near-cognate cases. The landscape describes switch I (Arg 58) distance to domain III (Ala375) of EF-Tu (DIII), describing the position of switch I with regards to the rest of the protein, and switch I (Arg 58) distance to the 3’ CCA end of the accommodating tRNA, describing the position of switch I with regards to accommodating tRNA. The simulations begin in the IB state and move towards an intermediate position of EF-Tu (Tu_int_), described previously^91^. Afterward three pathways describe if switch I proceed through the 1 – minor groove near the 3’-CCA end, 2 - major groove, or 3 - minor groove near the elbow during tRNA release before the tRNA can achieve the accommodated position.

As 91% of near-cognate trajectories follow globally similar pathways to cognate, we addressed if there are more subtle differences in the motion of these near-cognate aa-tRNAs, compared to cognate. Previously, we showed that EF-Tu’s switch I element traverses near either the tRNA major groove or minor grooves of the acceptor stem prior to aa-tRNA accommodation, with a narrow distribution^91^. To examine whether switch I of EF-Tu may traverse cognate and near-cognate aa-tRNA differently, we measured the reaction coordinates R_swI-DIII_, describing the position of switch I relative to domain III (DIII) of EF-Tu, and R_swI-CCA_, describing the position of switch I with respect to the 3’ CCA end of the accommodating aa-tRNA for both cognate and near-cognate aa-tRNA (Fig. 3a, b). Approximate free energy landscapes with these reaction coordinates describe the movement of switch I, beginning in the IB state, through CR and GA state, just prior to EF-Tu’s conversion to a semi-open intermediate conformation (Tu_int_) where switch I interacts with the acceptor stem of the aa-tRNA prior to accommodation (Fig. 3c, d).

As previously observed^91^, for cognate aa-tRNA, switch I has a narrow distribution of positions it can adopt either passing through the major groove or minor groove near the 3’-CCA end of the aa-tRNA (Fig. 3c inset). However, near-cognate aa-tRNA has a wider distribution of positions that it can adapt as it interacts with the aa-tRNA (Fig. 3d inset). This broader distribution reflected a higher probability of switch I of EF-Tu traversing the minor groove near the aa-tRNA elbow for near-cognate aa-tRNA. These findings suggest that near-cognate aa-tRNA can also adopt misaligned trajectories as it is being released from EF-Tu.

### Near-cognate tRNA further misaligns during accommodation

The accommodation step of proofreading selection entails movement of the aa-tRNA elbow domain and 3’-CCA end into the ribosome and away from EF-Tu, to achieve the AC (A/A) state from which peptide bond formation can occur. The rate-limiting aa-tRNA accommodation step^77^ is thus captured by the R_elbow_ reaction coordinate that describes the distance between the A- and P-site tRNA elbow domains^95^ and the R_cca-cca_ reaction coordinate that describes the distance between the center of mass of residue A76 at the extreme terminus of A- and P-site tRNA (Methods).

**Fig. 4:**
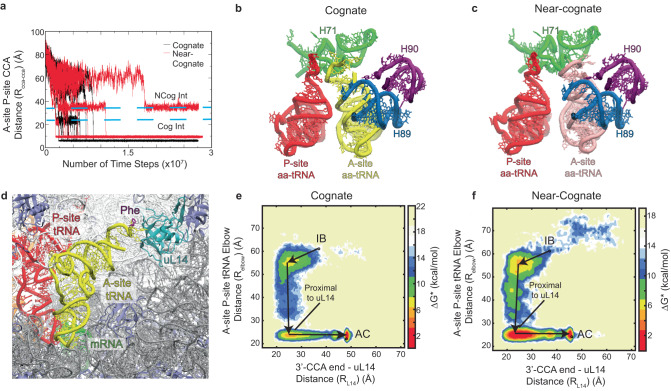
The Accommodation of cognate and misaligned near-cognate aa-tRNA. **a** The distance between the A76 for A and P site tRNA (reaction coordinate R_cca-cca_) over the course of the simulations. Intermediates around ~24 Å and ~37 Å can be observed for both cognate and near-cognate, respectively. Accommodation is considered completed in the simulations when the R_cca-cca_ distance reaches <10 Å. The average final R_cca-cca_ distance for cognate and near-cognate are 6.3 ± 0.3 and 9.6 ± 0.4 Å, respectively. **b** Intermediate conformation cognate aa-tRNA resolved from R_cca-cca_. In this conformation, both aa-tRNA interact with H89 (blue) and H71 (green) in proximity to H90 (purple). **c** Intermediate conformation of near-cognate aa-tRNA resolved from R_cca-cca_. The interactions between the aa-tRNA and H71 differ between cognate and near-cognate aa-tRNA. **d** Structural representation of the A-site aa-tRNA (yellow) interacting with ribosomal protein uL14. Free energy landscape of A-site and P-site tRNA elbow distance (R_elbow_) with respect to the distance between the 3’-CCA end and L14 (R_L14_) for cognate (**e**) and near-cognate (**f**). The near-cognate aa-tRNA that is misaligned in the A-site is stalled during its interactions with uL14.

Consistent with previous simulation efforts^97,98^, the 3’-CCA ends of both cognate and near-cognate tRNA did not begin to appreciably move towards the PTC until the aa-tRNA elbow domain achieved its accommodated position. At the accommodation endpoint, cognate aa-tRNA reached an observed R_cca-cca_ distance of <7 Å, positioning the nucleophile and electrophile on A- and P-site tRNA, respectively, within ~6 Å from each other from which peptide bond formation is likely to rapidly proceed (Fig. 4a, b; Supplementary Fig. 4; Supplementary Table 1). By contrast, near-cognate simulations ultimately reached more separated R_cca-cca_ values (ca. ~10 A) (Fig. 4a, c; Supplementary Fig. 4; Supplementary Table 1), indicative of codon-anticodon dependent misalignment of the A-site nucleophile during accommodation. Moreover, as has been reported previously^97,98^, both cognate and near-cognate aa-tRNA are accommodated via intermediate states along the R_cca-cca_ reaction coordinate (Fig. 4a).

Notably, the intermediate near-cognate aa-tRNA position was distinct from cognate aa-tRNA (R_cca-cca_ ~24 Å for cognate, ~37 Å for near-cognate) (Fig. 4a). In both cases, the acceptor stem of cognate and near-cognate aa-tRNAs interacted with helix 89 and 90 of the 23S rRNA (H89, H90) of the accommodation corridor^97,98^. In addition to these H89/H90 interactions, the 3’ CCA end of the near-cognate aa-tRNA interacted with helix 71 (H71) of the 23S rRNA in the LSU^97,98,111^ (Fig. 4b, c), where mutations have been evidenced that increase stop-codon readthrough decoding errors^112^. The observed distinctions in aa-tRNA passage through the evidenced intermediate aa-tRNA positions were captured by plotting the R_elbow_ reaction coordinate against an R_L14_ reaction coordinate, which measures the distance between the tRNA 3’-CCA end and the center of mass of ribosomal protein uL14 immediately proximal to H71 (Fig. 4d–f). These data illustrated both the near-cognate aa-tRNA passage of the GA state, marked by an initial accumulation at 60 Å separated R_elbow_ distances, as well as its slower rate of accommodation, marked by the accumulation of R_L14_ values at ~25 Å, where the 3’-CCA end interacts with elements of the accommodation corridor. Consequently, the observed frequency of cognate and near-cognate aa-tRNA accommodation attempts were similar (~2 μs^−1^); however, the pathways and barrier heights differed between cognate and near-cognate tRNAs (Supplementary Material; Supplementary Table 1). Taken together with the previously determined accommodation barrier for cognate tRNAs (~9–13 k_B_T)^96^, we estimate an average accommodation rate in the cognate case of ~5 s^−1^ in approximate agreement – albeit slower – with those determined biochemically for cognate aa-tRNA^77,87^. For near-cognate aa-tRNA to exhibit significantly lower rates of accommodation (ca. 100-fold reduced) the barrier would have to be ~5 k_B_T larger than cognate. We attribute the additional barrier height to near-cognate aa-tRNA accommodation to the non-canonical codon-anticodon base pairing, and the misaligned positions it gives rise to, which reduce the probability of successfully reaching the endpoints of initial selection of proofreading that leads to productive chemistry.

### Fully accommodated near-cognate tRNAs remain misaligned for peptide bond formation

**Fig. 5:**
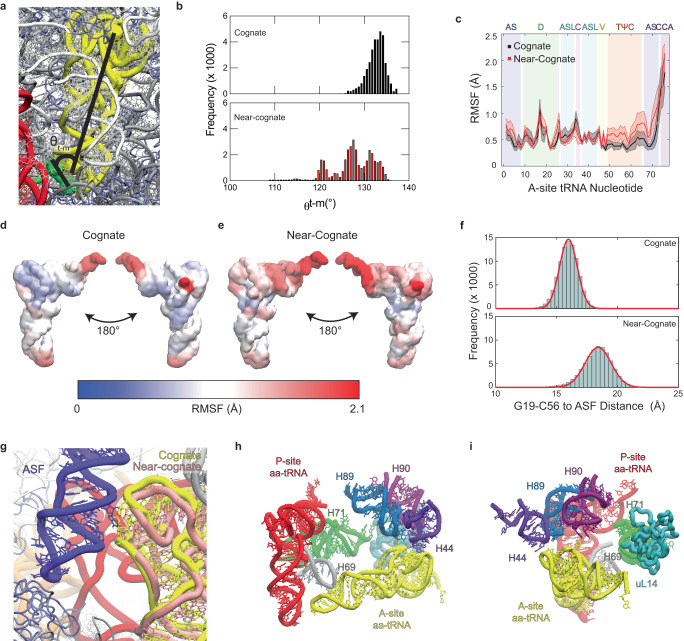
Near-cognate aa-tRNA is misaligned in the A site of the ribosomes. **a** Measurement of θ_t-m_ angle between the mRNA codon (between the center of mass of residue 6 and 8) and the length of the tRNA (between the center of mass of the anticodon and U60). **b** Angles between the vector produced between position 1 and 3 of the codon and the vector between the aa-tRNA anticodon stem loop and elbow, after the simulation has reached the AC state. **c** Root Mean Squared Fluctuation (RMSF) of the tRNA during simulations of initial selection and aa-tRNA accommodation. Near-cognate aa-tRNA display enhanced RMSF at the TΨC loop and acceptor stem. The solid line indicates the average RMSF for 20 simulations with the shaded area representing the standard deviation. Structural mapping of accommodating tRNA RMSF during tRNA selection for (**d**) cognate and (**e**) near-cognate aa-tRNA. **f** Distance between the G19-C56 nucleotide base-pair (center of mass of G19 and C56 O3’ atoms) of the accommodating aa-tRNA measured relative to the A-site finger (O3’ of nucleotide C898). The average distance for cognate and near-cognate aa-tRNA is 16.0 Å and 18.4 Å. **g** Representative positions of cognate (yellow) and near-cognate (pink) in regard to the ASF (blue) of the ribosome. Structural representation of cognate aa-tRNA (yellow) at the beginning of the accommodation corridor, composed of H89 (blue), H90 (pink), H71 (green), H44 (purple), H69 (gray), uL14 (cyan), (**h**) view from the central protuberance (cognate), (**i**) view from the accommodation corridor entrance (cognate).

To investigate how near-cognate aa-tRNA accommodation leads to different R_cca-cca_ values, we investigated whether we could evidence aa-tRNA distortions within the A site. For this analysis, we assessed the orientation of the tRNA in the A site by quantifying the angle between the vector derived from mRNA-codon nucleotides 1 and 3 and the vector between the aa-tRNA elbow and anticodon (θ_t-m_) domains (Fig. 5a). While the cognate aa-tRNA formed an average angle of 132°, near-cognate adopted three different populations with average angles of 121, 127, and 131° (Fig. 5b; Supplementary Table 2). This finding suggests that near-cognate codon-anticodon interactions lead to a broadening of the distribution of near-cognate positions within the LSU A site during proofreading.

To investigate the flexibility of aa-tRNA within the A site, we measured the root mean square fluctuation (RMSF) of each O3’ atom in accommodated cognate and near-cognate tRNA molecules (Fig. 5c–e). While the majority of both tRNAs exhibited similar flexibility, in near-cognate tRNA we observed an increase in the localized flexibility of nucleotides C49-C72 of the TΨC-loop and acceptor stem (Fig. 5c–e). Congruent with this increased flexibility playing a role in the tRNA selection process, this region of near-cognate showed reduced flexibility in the presence of GEN and NEO (Supplementary Fig. 5). Related to this finding, we observed that the tRNA elbow domains of near-cognate aa-tRNAs were situated at different positions, consistent with misalignment of near-cognate aa-tRNA relative to the A-site finger, a region whose flexibility fluctuates during subunit rotation and tRNA accommodation (Fig. 5f, g)^73,85,113–115^. In the cognate AC (A/A) state, an extruded adenosine base within the A-site finger stacks with the universally conserved G19-C56 base pair within the aa-tRNA elbow^85^, which are not observed in near-cognate aa-tRNA positioning in near-cognate simulations (Fig. 5g).

As the altered aa-tRNA position observed for near-cognate aa-tRNA correlated with an increased distance between the nucleophile and electrophile within the PTC, we employed distinct distance measurements to triangulate the position of the tRNA within the A site (Table 1), including specific regions of the tRNA to closest to rRNA and ribosomal protein elements (Fig. 5h, i). These measurements revealed that distinctions in near-cognate aa-tRNA positioning manifest in the acceptor stem, indicated by distances to H90, and are then amplified to the position of the 3’ CCA end of the aa-tRNA (Table 1). Hence, perturbed near-cognate aa-tRNA geometries with respect to the mRNA amplify through the tRNA body to manifest in altered positioning of the 3’ CCA end bearing the nucleophile for peptide bond formation.

**Table 1:** Distances between ribosomal elements and regions of the accommodated aa-tRNA in the A site

	H89 – aa-tRNA elbow (Å)	H69 – anticodon stem (Å)	H90 – acceptor stem (Å)	H44 – aa-tRNA elbow (Å)	H71 – 3’ CCA end (Å)	L14 – 3’ CCA end (Å)
Cognate aa-tRNA	31.3 ± 4.3	18.2 ± 2.7	29.4 ± 5.2	31.5 ± 7.0	34.4 ± 2.1	47.4 ± 2.2
Near cognate aa-tRNA	31.1 ± 4.6	19.5 ± 2.4	24.8 ± 6.2	29.7 ± 5.2	30.5 ± 2.4	43.6 ± 1.8

Distances are only measured after the aa-tRNA is considered accommodated and are averaged over 40 simulations, each with 1 × 10^8^ timesteps. Reported are the culminated average distance from each simulation and culminated standard deviation after error propagation.

### Antibiotics (Peptidyltransferase inhibitors) induce aa-tRNA misalignments

**Fig. 6:**
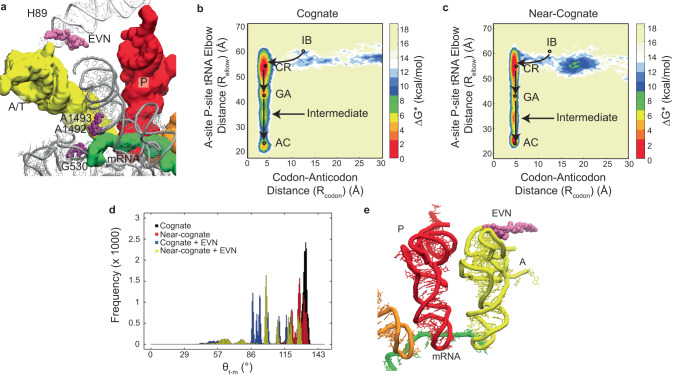
EVN induces near-cognate-like accommodation trajectories for aa-tRNA. **a** Structural representation of EVN (brown) bound to the H89 (white) of the 50 S ribosomal subunit, with A/T (yellow) and P site (red) tRNA, mRNA (green), and 16 S (grey) G530, A1492, and A1493 highlighted (purple). Free energy landscapes of the accommodation pathway of cognate (**b**) and near-cognate (**c**) tRNA into the ribosomal A-site described by the R_elbow_ and R_codon_ reaction coordinates. **d** The angle of tRNA with respect to the A-site codon (θ_t-m_) of cognate and near-cognate aa-tRNA in the presence and absence of EVN during proofreading. **e** EVN interacting with the major groove of tRNA during the accommodation pathway, inducing compression of the tRNA.

As we described above, 4,5- and 4,6-linked aminoglycosides can alleviate misalignment signatures of near-cognate aa-tRNA. We, therefore, asked if antibiotics that inhibit cognate aa-tRNA selection promote tRNA misalignment. First, we investigated EVN which prevents accommodation of aa-tRNA by providing a steric barrier in the accommodation corridor^53,116^ through structure-based simulations for cognate and near-cognate aa-tRNAs with EVN bound to H89 (Fig. 6a). Consistent with EVNs binding site with the LSU accommodation corridor, EVN exerted its steric impacts on aa-tRNA during its passage through the LSU accommodation corridor while having no observable impact on events prior to the formation of GA-like states (Fig. 6b, c, e). Consequently, both cognate and near-cognate aa-tRNAs exhibited prolonged intermediate positions during accommodation corridor passage (Fig. 6b, c), which were distinct from those evidenced during cognate aa-tRNA selection in the absence of antibiotics, but consistent with those for misaligned near-cognate aa-tRNA (Fig. 6d; Supplementary Table 2)^92^. Our simulations therefore suggest that EVN favors cognate aa-tRNA traversing accommodation trajectories, and adopting additional aa-tRNA angles of approach within the accommodation corridor, that are similar to those transited by near-cognate aa-tRNA (Fig. 6d; Supplementary Table 2). Visualization of the simulations revealed that EVN promotes altered cognate tRNA positions and delays accommodation by sterically interacting with the major groove of the tRNA acceptor stem and TΨC loop, which is ultimately overcome by compression of the tRNA elbow domain, allowing passage into the LSU PTC (Fig. 6e).

**Fig. 7:**
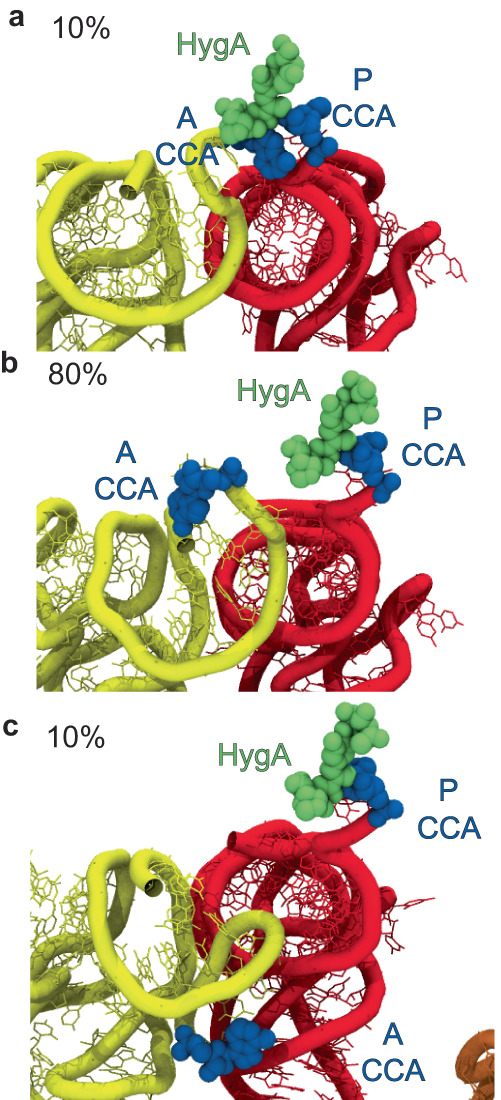
HygA promotes the 3’ CCA end of tRNA to accommodate into positions similar to near-cognate aa-tRNA. Conformations of cognate tRNA during accommodation in the presence of HygA (green) (A76 highlighted in blue), where the 3’-CCA end of the aa-tRNA achieves (**a**) stacking with HygA in the fully accommodated position (10% of simulations), (**b**) cannot properly engage with the PTC due to a steric hindrance of HygA (80% of simulations), or (**c**) is mispositioned entirely due to steric hindrance of HygA.

Second, X-ray crystallography studies indicate that the antibiotic HygA alters the positioning of the terminal adenosine of the accommodating aa-tRNA molecule so as to prevent peptide bond formation^64^. Structure-based simulations with HygA bound adjacent to the PTC revealed that while the aa-tRNA can reach fully accommodated positions (Supplementary Fig. 6), both the cognate and the near-cognate aa-tRNA 3’-CCA ends exhibited altered positions (Fig. 7a–c). Consistent with X-ray crystallography^64^, we observed A76 stacking with HygA (Fig. 7a), as well as evidence that the aa-RNA 3’-CCA end can fold back on itself in multiple orientations (Fig. 7b, c). Hence, for both EVN and HygA, steric clashes that arise from drug occupancy in their respective binding sites can misalign distinct elements of the tRNA molecule to effectively delay engagement of the tRNA 3’-CCA end with the PTC^64,117^, likely contributing to premature aa-tRNA release and additional tRNA selection events that result in non-productive energy expenditure^66^.

## Discussion

**Fig. 8:**
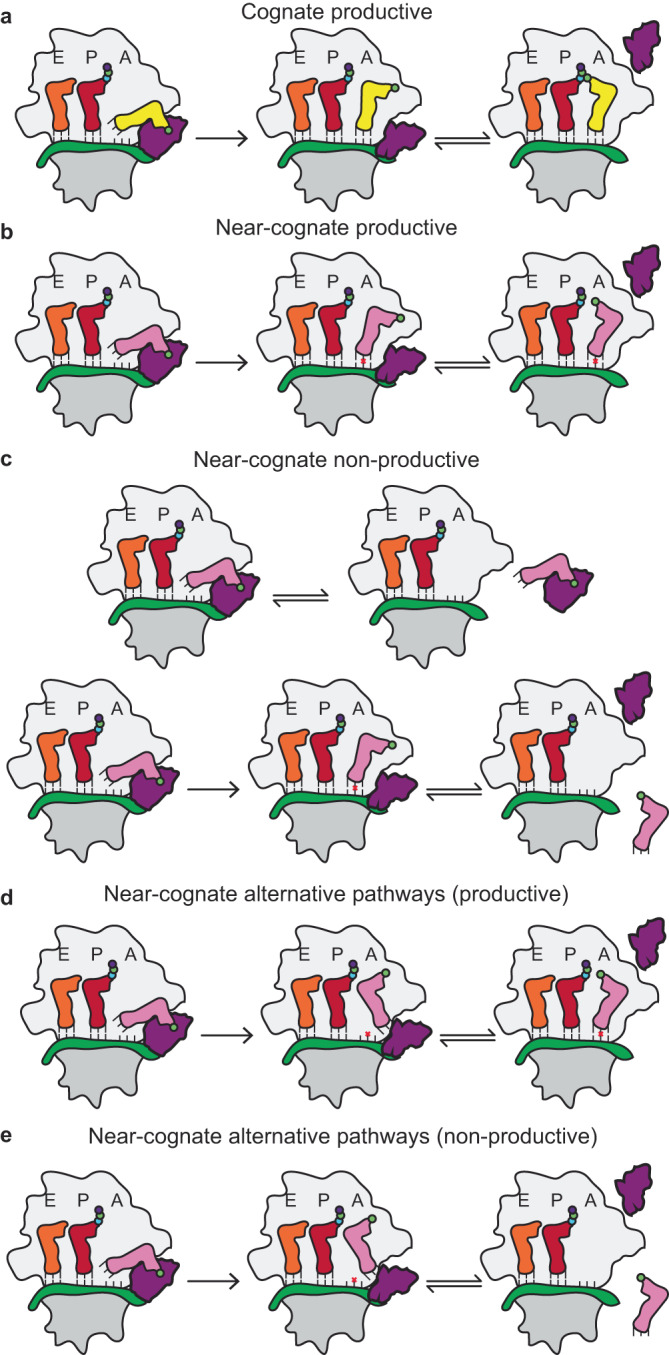
Observed accommodation trajectories for near-cognate aa-tRNA during tRNA selection. **a** Productive cognate aa-tRNA selection pathway where initial selection and proofreading lead to an accommodated cognate aa-tRNA. **b** Productive near-cognate aa-tRNA selection pathway where initial selection and proofreading lead to an accommodated near-cognate aa-tRNA. **c** Non-productive near-cognate aa-tRNA selection where codon-anticodon interactions don’t form and lead to aa-tRNA rejection or aa-tRNA is misaligned during proofreading and is rejected. **d** Near-cognate productive alternative pathways where the aa-tRNA attempts to enter the ribosome elbow first in the absence of codon anticodon interactions, which form subsequently. **e** Near-cognate non-productive alternative pathways where the lack of codon-anticodon interactions facilitates aa-tRNA dissociation after the elbow domain has entered the ribosome.

Our findings support a model in which the tRNA selection process is governed by geometric considerations related to the alignment of aa-tRNA relative to the catalytic centers on the LSU, such as the site where EF-Tu catalyzes GTP hydrolysis^92^ and the peptidyltransferase center (Fig. 8a, b, c; Supplementary Figs. 7a–c). Due to the reduced enthalpic contribution of near-cognate codon-anticodon interactions, the aa-tRNA becomes misaligned and fails to productively navigate its way in the constrained, fully accommodated position where peptide bond formation occurs. This negatively impacts the probability of forward, productive enzymatic reactions in the presence of near-cognate aa-tRNA. These findings indicate that the ribosome has evolved the accommodation corridor as an environment at optimum crowding conditions for cognate aa-tRNA selection, as molecular crowding similarly can impact RNA and protein folding thermodynamics and kinetics^118–121^. In the event that near-cognate aa-tRNA does undergo peptide bond formation, the mechanical force imposed on the tRNA from the nascent peptide^122^ could contribute to properly positioning the tRNA for subsequent translocation. The model we propose states that relatively minor distinctions in the nature of mRNA codon-tRNA anticodon pairing for near-cognate aa-tRNA fail to appropriately engage the anticodon stem loop so as to allow a broad range of aa-tRNA approach vectors as it enters into the A site (Fig. 8d, e). This fits within the framework of tRNA selection where aa-tRNA can undergo productive selection, non-productive tRNA selection, or achieve misaligned positions (Fig. 8)^77,80^. Consistent with this view, we found that antibiotics both negatively and positively affect the tRNA selection mechanism by either causing or reducing misalignment, depending on the specific antibiotic.

The structure-based simulations used to model the tRNA selection pathway of cognate and near-cognate aa-tRNA do have some limitations. First, the contacts identified in the fully AC position are defined as the energetic minima, resulting in the system sampling configurations until it achieves the AC conformation. Given this framework, we do not observe direct interconversion between the GA and AC conformations in these simulations. Furthermore, we can only characterize the steric constraints of the accommodation of tRNA selection as the electrostatic effects, water molecules, and ions are not considered in these simulation types. Regardless, our simulations indicate that the crowding environment imposed on the tRNA during the selection process is sufficient to promote distinct pathways of cognate and near-cognate aa-tRNA during selection. After the molecular simulations were performed a cryo-EM structure of near-cognate aa-tRNA in an AC position was observed^73^. The cryo-EM density supports our findings that the near-cognate aa-tRNA is misaligned during tRNA selection and that the elbow region of the tRNA is dynamic in the AC position.

These structure-based simulations indicate that the near-cognate aa-tRNA has a reduced probability for the tRNA to form stable or immediate codon-anticodon interactions with the mRNA as indicated by the ~9% of simulations that attempt to adopt an alternative pathway of motion during selection (Fig. 2c). Under this framework the near-cognate aa-tRNA may have an increased probability of engaging the frame at the +1 position similar to suppressor tRNA that utilizes a 4 base-pair anticodon^123,124^.

The present near-cognate simulations have a single G•U mismatch in the middle base-pair of the codon-anticodon base pair. As mismatches at the middle nucleotide have the greatest impact on decoding accuracy^125^ it is likely that mismatches at this position have the largest impacts on tRNA alignment^82^. The broad tolerance of wobble-position mismatches observed in nature suggests that the impact of mismatches may be particular to the first and second positions. More disruptive mismatches at the middle position than the one presently explored or elsewhere in the codon-anticodon pair (e.g. purine•purine mismatches), would be expected to further enhance the likelihood of aa-tRNA misalignment during tRNA selection. Conversely, molecules, such as NEO and GEN which bind to H44 appear to modulate aa-tRNA misalignment. It is therefore interesting to determine whether other molecules that interact with H44 such as spermidine may also impact tRNA alignment^126^. Similarly, modifications to either the mRNA or tRNA would alter the basis of the codon-anticodon interactions^127^, influencing the alignment of aa-tRNA during selection.

A key feature that this adds to the kinetic proofreading framework of tRNA selection is that the relatively long lever arm of the tRNA molecule (70 Å from anticodon to elbow) geometrically amplifies, over distance, the relatively small deviations in the codon-anticodon pair at the decoding center (Supplementary Fig. 7). Within this framework the decoding nucleotides of A1492, A1493, and G530 recognize the base-pairs within the codon-anticodon minihelix, GTP hydrolysis is triggered and aa-tRNA begins the trajectory into the A site. Originating at non-ideal Watson-Crick base-pairs near-cognate aa-tRNA becomes misaligned during its trajectory into the A site. This is supported by kinetic data showing that accommodation of near-cognate aa-tRNA into the A site is ~75 fold slower than cognate^18^. A misaligned aa-tRNA would form more contacts with the accommodation corridor, providing more opportunity for reversible fluctuations observed by smFRET^77^, during the trajectory into the A site, reducing the rate of the entire process. As such, the misaligned aa-tRNA and the amplifications of the deviations reside within kinetic proofreading and contribute to the accuracy of decoding. Intriguing is the observation that modifications to tRNA nucleotides can increase accuracy during decoding^128,129^. Under a misalignment model these modifications, which add steric bulk to the tRNA and additional charges, would narrow the already confined space of the accommodation corridor. Thus, these modifications would decrease the ability for near-cognate tRNA traverse proofreading efficiently.

The mismatches in the codon-anticodon helix can lead to distortions in the codon-anticodon minihelix, either by widening, shortening, or shifting nucleobases to a minor or major groove providing the origin of the misalignment^33^. In an understanding of the physical chemistry of decoding, we consider that the G-U mismatch base-pair is the most often observed mismatch that proceeds through initial selection^7,128^, as the nucleotides adopt a Watson-Crick like base-pair. The position of this base-pair results in minimal perturbation at the codon-anticodon site yet is still kinetically unfavored to accommodate compared to cognate. If A1492, A1492, and G530 cannot distinguish the codon-anticodon interactions during proofreading the length of the tRNA provides an opportunity for selection. Unintuitively, a U-G base-pair that also retains Watson-Crick like geometry is even less observed^102,130^, indicating that codon-anticodon geometry during decoding is not the universal selector for tRNA. Positioning the pyrimidine in the mRNA would ostensibly not shift the minihelix further than a G-U pair. The physical consequence could reside in a U-G base-pair providing a signal that is amplified differently so that the CCA end shifts further towards the steric interactions of the accommodation corridor, correlating the observed G-U / U-G mismatch accuracy differences. This misalignment and amplification model provides a physical rationale for kinetic proofreading models of tRNA selection^13–16^ and a molecular basis for tRNA selection fidelity. Importantly, it also parsimoniously explains why both tRNAs and the ribosome have remained large across evolution (Supplementary Fig. 8), despite the associated metabolic and energetic costs associated with their biogenesis.

## Methods

### 70S•EF-Tu•nucleotide•tRNA•mRNA model building

Models of the mRNA-programmed 70S ribosome in complex with the EF-Tu•GTP•tRNA ternary complex with cognate or near-cognate codon-anticodon interactions in the A site were derived from previously resolved cryo-EM structures (PDB ID: 5UYK, 5UYL, 5UYM, 5UYN, 5UYP, and 5UYQ)^79^. That is, the initial starting configuration (IB) of the simulation, coordinates for the ribosomal proteins, ribosomal RNA, EF-Tu, mRNA, peptidyl tRNA, cognate aminoacyl tRNA (tRNA^Phe^), and near-cognate aminoacyl tRNA (tRNA^Lys^) were all taken directly from the published cryo-EM structures (73). Either GTP or GDP along with coordinated Mg^2+^ was added to each system by aligning EF-Tu with crystal structures of EF-Tu containing bound nucleotide (PDB ID: 1EFT and 1EFC respectively)^131,132^. All alignments were performed with VMD 1.9.2^133^. The GDPNP from 1EFT was converted to GTP by replacing the nitrogen separating the γ and β phosphates with oxygen. Each tRNA was aminoacylated according to the proper chemistry. Force field parameters corresponding to the native state in the structure-based simulations (i.e., the fully accommodated tRNA conformations (A/A)) were obtained by aligning the GA state with the cryo-EM structure of aa-tRNA in the post-accommodated state (PDB ID: 4V66). For these force field parameters, the coordinates of the accommodated tRNA were added to GA state and the coordinates of the previous tRNA were removed. While the structure model of the near cognate tRNA was taken directly from the cryo-EM structure (i.e., the pre-A/T state), the force field parameters for the native state (i.e., the A/A state), were obtained by converting the A site tRNA in the near-cognate AC state to the sequence of tRNA^Lys^ with the Swapna package in UCSF Chimera^134^.

Models containing EF-Tu in the open or GDP conformation were constructed by aligning and replacing EF-Tu with the crystal structure of EF-Tu in the GDP conformation (PDB ID: 1EFC). Coordinates for GEN and NEO in models were adapted from aligned structures of the 70S bound to the antibiotics (PDB ID: 4V53 and 4WOI, respectively)^72,135^. Nucleotides A1492 and A1493 of the 16S rRNA along with A1912 of the 23S were also adapted from these structures after alignments for the AC state. Similarly, coordinates of EVN and HygA were derived by aligning the 70 S with the structures of EVN and HygA bound (PDB ID: 5KCS and 5DOY, respectively)^53,64^.

Before performing molecular simulation, the energy of each of the models was minimized using the steepest descent approach in GROMACS v4.5.4 with AMBERFF99S force fields^136–140^. Minimizations were performed in an explicit solvent system within TIP3P water molecules with 100 mM KCl, resolved Mg ions, and 7 mM MgCl_2_ for 10,000 steps each. For the minimization of 70S with A/A site tRNA an additional round of energy minimization was employed, minimizing the energy of the A/A site tRNA_,_ followed by water molecules, and then the entire system, was minimized sequentially.

### Structure-based simulations

All atom structure-based models were constructed using Smog-2.1.0^103^. Simulations were performed as using a single gaussian basin potential, previously utilized^141^, at a temperature of 0.5ε with Langevin dynamics, where ε is reduced units. The native contact potential (C_ij_) is defined by:

1 $${C}_{W}\left({r}_{{ij}},{r}_{{ij},0}\right)=\left(1+{\left(\frac{{\sigma }_{{NC}}}{{r}_{{ij}}}\right)}^{12}\right)\left(1+W\left({r}_{{ij}},{r}_{{ij},0}\right)\right)-1$$

where,

2 $$W\left({r}_{{ij}},{r}_{{ij},0}\right)=-\exp \left[\frac{-{\left({r}_{{ij}}-{r}_{{ij},0}\right)}^{2}}{2{\sigma }^{2}}\right]$$

In this framework σ_NC_ = 2.5 Å and is the excluded volume size, r_ij_ is the distance between atoms i and j, r_0_ are the distances of these atoms in the A/A configuration, and σ is the width of the gaussian well set to a depth of −1. For each condition, 100 simulations were performed with random initial velocities and a step size of 0.005 for 250 000–750 000 t_ru_. The potential (V_ij_) for each simulation was defined by:

$${V}_{{ij}}=\mathop{\sum}\limits_{{bonds}}\frac{{\varepsilon }_{r}}{2}{({r}_{i}-{r}_{i,o})}^{2}+\mathop{\sum}\limits_{{angles}}\frac{{\varepsilon }_{\theta }}{2}{({\theta }_{i}-{\theta }_{i,o})}^{2}$$

$$+\mathop{\sum}\limits_{{impropers}}\frac{{\varepsilon }_{\chi i}}{2}{({\chi }_{i}-{\chi }_{i,o})}^{2}+\mathop{\sum}\limits_{{plainar}}\frac{{\varepsilon }_{\chi p}}{2}{({\chi }_{i}-{\chi }_{i,o})}^{2}$$

$$+\mathop{\sum}\limits_{{backbone}}{\varepsilon }_{{BB}}{F}_{D}({\phi }_{i}-{\phi }_{i,o})+\mathop{\sum}\limits_{{sidechains}}{\varepsilon }_{{SC}}{F}_{D}({\phi }_{i}-{\phi }_{i,o})$$

3 $$+\mathop{\sum}\limits_{{contacts}}{\epsilon }_{C}{C}_{W}\left({r}_{{ij}},{r}_{i,j,0}\right)+\mathop{\sum}\limits_{{non}-{contacts}}{\varepsilon }_{{NC}}{\left(\frac{{\sigma }_{{NC}}}{{r}_{{ij}}}\right)}^{12}$$

where,

4 $${\varepsilon F}_{D}\left(\phi \right)=\varepsilon \left(1-\cos \phi \right)+\frac{\varepsilon }{2}\left(1-\cos 3\phi \right)$$

and $${\varepsilon }_{r}$$ = 50$${\varepsilon }_{0}$$, $${\varepsilon }_{\theta }$$ = 40$${\varepsilon }_{0}$$, $${\varepsilon }_{\chi i}$$ = 10$${\varepsilon }_{0}$$, $${\varepsilon }_{\chi p}$$ = 40$${\varepsilon }_{0}$$, $${\varepsilon }_{{NC}}\,$$= 0.1$${\varepsilon }_{0}$$,$${\sigma }_{{NC}}$$ = 2.5 Å, and $${\varepsilon }_{0}$$ = 1

All bond distances, angles, and non-bonded contacts of the AC conformation were set as the native state. The simulation was initiated in pre-A/T state and approached the AC state through thermal fluctuations. As these simulations cannot resolve chemical reactions such as GTP hydrolysis this step of aa-tRNA selection was not included in the simulations. However, by employing a system where the non-bonded contacts between the mRNA and tRNA are stronger than the accommodated tRNA position, we ensure that initial selection (formation of codon-anticodon base-pairs) occurs prior to tRNA accommodation. Therefore, a discrepant non-bonded contact strength setup allows us to mimic the gating between initial selection and accommodation provided by the GTP-hydrolysis step. Specifically, contacts between mRNA and aa-tRNA were scaled by 0.8 while contacts between aa-tRNA and the ribosome were scaled by 0.4, 0.6, or 0.8 to ensure base-pairing between the codon-anticodon occurs prior to reversible excursions of aa-tRNA^77,97^, consistent with smFRET^76,77^. A contact weighting of 0.8 for mRNA-tRNA interactions reflects the stability of Watson-Crick base-pairs compared to the non-Watson Crick interactions that are formed between the tRNA and the ribosome in the AC state. These simulations produce trajectories consistent with smFRET and cryo-EM data consisting of aa-tRNA that follow the reversible tRNA selection path of IB-CR-GA-AC^73,77–79^. Although not utilized in the molecular simulations from this study, a user could introduce “non-native” contacts in the structure-based potential^142,143^. “Non-native” contacts can be beneficial to stabilize an intermediate or a simulation endpoint. Introducing non-native contacts needs to be performed cautiously as the inappropriate addition of non-native contacts could lead to artificially stabilized conformations and could produce artifacts within the molecular simulations.

### Reaction coordinate calculations

The reaction coordinates R_elbow_ monitoring tRNA accommodation was used as previously^95,144^. This reaction coordinate is the distance between the O3’ atoms of U8 in the P-site tRNA and U60 in the A-site tRNA and describes the barrier for aa-tRNA accommodation during interactions with H89 (Supplementary Fig. 9). R_codon_ was measured between the N3 of the wobble position C in the mRNA and the N1 atom of G34 in the A-site tRNA for cognate codon-anticodon interactions (Supplementary Fig. 9). For near-cognate codon-anticodon interactions, R_codon_ was measured between the N1 atom of the A in the wobble position of the mRNA and the N3 atom of U34 in the A-site tRNA. R_codon_ was used to reveal the timing and barrier for codon anticodon interactions. R_cca-cca_ is defined by the distance between the center of mass of A76 for both the A-site and P-site tRNA^97^. As the 3’-CCA end is the final step in aa-tRNA accommodation, the reaction coordinate describes the barrier of the 3’-CCA accommodation through its interactions with the ribosomal A loop, H89, and H90 (Supplementary Fig. 9)^97^. R_L14_ is defined as the distance between ribosomal protein uL14 and the 3’-CCA end of the A-site tRNA during accommodation. This reaction coordinate describes the barrier for aa-tRNA accommodation as the 3’-CCA end interacts with ribosomal protein uL14. R_swI-DIII_ is defined as the distance between Arg 58 of switch I of EF-Tu and Ala 375 of domain III of EF-Tu, describing the position of switch I relative to EF-Tu during conformational rearrangement. R_swI-CCA_ is the distance between Arg 58 of switch I and the center of mass of the 3’ CCA nucleotides of the accommodating aa-tRNA, describing the position of switch I relative to the accommodating aa-tRNA.

Boltzmann weighted approximate free energy landscapes comparing reaction coordinates were calculated using Eq. 5 found in the g_sham package in GROMACS v4.5.4^138–140^. In equation 5, ΔG represents the approximate free energy, k_B_ is the Boltzmann coefficient, T is the temperature (300 K), P(x_i_) is the probability density function of being at state i obtained from a histogram of the MD data, and P_max_(x) is the maximum probability of the most observed state^91,145^.

5 $$\Delta G={-k}_{B}T{{{{\mathrm{ln}}}}}{\left(\frac{P\left({x}_{i}\right)}{{P}_{\max }\left(x\right)}\right)}^{2}$$

### Distance measurements

To triangulate the position of the accommodated aa-tRNA, several distances were measured between the large ribosomal subunit and the accommodating aa-tRNA. The distance between H44 of the LSU and the aa-tRNA elbow was measured between the O3’ atom of A1095 of the 23S rRNA and C57 of the aa-tRNA. The distance between H69 and the anticodon stem was measured between the O3’ atom of C1914 of the 23S rRNA and A38 of the aa-tRNA. The distance between H90 and the acceptor stem was measured between the O3’ atom of G2536 of the 23S rRNA and C3 of the aa-tRNA. The distance between H89 and the aa-tRNA elbow was measured between the O3’ atom of G2472 of the 23S rRNA and C56 of the aa-tRNA. The distance between H71 and the 3’ CCA end of the aa-tRNA was measured between the O3’ atom of A1953 of the 23S rRNA and A76 of the aa-tRNA. The distance between uL14 and the 3’ CCA end of the aa-tRNA was measured from the CA atom of Asp 56 of uL14 and the O3’ atom of A76 of the aa-tRNA.

Average distance measurements were determined using a single gaussian distribution fit as defined by:

6 $${counts}={Ae}\frac{-{\left(x-{\bar{x}}_{a}\right)}^{2}}{2{s}_{a}^{2}}$$

where *A* is the peak of the distribution, ${\bar{x}}_{a}$ is the average value of the distance, and ${s}_{a}$ is the standard deviation for the population.

### tRNA-mRNA angle calculations

The angle between the tRNA and mRNA (θ_t-m_) (Fig. 5A) was defined by:

7 $${\theta }_{t-m}={{\arccos }}\frac{({v}_{m}\cdot {v}_{t})}{\left|{v}_{m}\right|\left|{v}_{t}\right|},$$

where *v*_*m*_ is the vector produced between the centers of mass of residues 6 and 8 of the mRNA (or positions 1 and 3 in the codon), and *v*_*t*_ is the vector between the center of mass of the anticodon (residue 34 to 36) of the tRNA with the center of mass of tRNA residue U60.

### Convergence of simulations

Convergence of the simulations was determined as in Vaiana and Sanbonmatsu 2009^81^ by calculating the pointwise RMSD between successive free energy landscapes, as a function of greater and greater sampling. The pointwise RMSD convergence metric is defined by

8 $${Conv}\left(t\right)=\sqrt{\frac{{\sum }_{i,j}{(\Delta {G\left(i,j\right)}_{t}- \Delta {G\left(i,j\right)}_{t0})}^{2}}{N}},$$

where $\triangle {G\left(i,j\right)}_{t}$ is the approximate free energy of R_elbow_ and R_codon_ after sampling to time t and $\triangle {G\left(i,j\right)}_{t0}$ is the approximate free energy of R_elbow_ and R_codon_ after sampling to time *t*_*0*_ (i.e., 1000 frames of simulation), from Eq. 5, and N is the number of grid points on the free energy landscape. Simulations were considered to have converged when *Conv*(t) plateaus. Additionally, the statistical fluctuations of the energy landscapes ($\zeta \left(t\right)$) were determined to approximate convergence by:

9 $$\zeta \left(t\right)=\sqrt{\frac{{\sum }_{i,j}{(\Delta {G\left(i,j\right)}_{t}- \Delta {G\left(i,j\right)}_{t- \Delta t})}^{2}}{N}}$$

### Reporting summary

Further information on research design is available in the Nature Portfolio Reporting Summary linked to this article.

### Supplementary information

Supplementary Information

Description of Additional Supplementary Files

Supplementary Movie 1

Supplementary Movie 2

Supplementary Movie 3

Reporting Summary

### Source data

Source Data

## Data Availability

The authors declare that the data supporting the findings of this study are available within the paper and its supplementary information files. Source data are provided with this paper.
