# Supplementary material for: Geometric alignment of aminoacyl-tRNA relative to catalytic centers of the ribosome underpins accurate mRNA decoding
Source: Nat Commun. 2023 Sep 11;14:5582. doi: 10.1038/s41467-023-40404-9 (PMC10495418; doi:10.1038/s41467-023-40404-9)
Supplement: Supplementary file 1 — Supplementary Information [file 41467_2023_40404_MOESM1_ESM.pdf]

Supplementary Information

Geometric alignment of aminoacyl-tRNA relative to catalytic centers of the ribosome underpins accurate mRNA decoding

AUTHORS:

Dylan Girodat[1,2], Hans-Joachim Wieden[3], Scott Blanchard[4*], Karissa Y. Sanbonmatsu[1,5*]

AFFILLIATIONS:

[1]Theoretical Biology and Biophysics, Theoretical Division, Los Alamos National Laboratory, Los Alamos, NM 87545, USA.

[2]Current Address: Department of Chemistry and Biochemistry, University of Arkansas, Fayetteville, AR, 72701, USA.

[3]Department of Microbiology, University of Manitoba, Winnipeg, MB R3T 2N2, Canada.

[4]Department of Structural Biology, St. Jude Children's Research Hospital, Memphis, TN 38105, USA.

[5]New Mexico Consortium, Los Alamos, NM 87545, USA.

*Correspondence to: kys@lanl.gov, scott.blanchard@stjude.org

SUPPLEMENTARY METHODS

Model Building

Published cryo-EM structures were used as the initial structures for model generation of the IB, CR, and GA states (PDB ID: 5UYK – IB, 5UYL – CR, 5UYM – GA, 5UYN – near-cognate IB, 5UYP – near-cognate CR, and 5UYQ – near-cognate GA) [1]. Either GTP or GDP along with coordinated Mg^{2+} was added to each system by aligning EF-Tu with crystal structures of EF-Tu containing bound nucleotide (PDB ID: 1EFT and 1EFC respectively) [2,3]. All alignments were performed with VMD 1.9.2 [4]. The GDPNP from 1EFT was converted to GTP by replacing the nitrogen separating the γ and β phosphates with an oxygen. The fully accommodated tRNA conformations (A/A) were constructed by aligning the GTPase activated state with the cryo-EM structure of aa-tRNA in the post-accommodated state (PDB ID: 4V66). The coordinates of the accommodated tRNA were added to the GTPase activated state and the coordinates of the previous tRNA were removed. The A site tRNA in the near-cognate A/A state was manually converted to the sequence of $tRNA_{Lys}$ with the swapna package in UCSF Chimera [5].

Models containing EF-Tu in the open or GDP conformation were constructed by aligning and replacing EF-Tu with the crystal structure of EF-Tu in the GDP conformation (PDB ID: 1EFC). Coordinates for GEN and NEO in models were adapted from aligned structures of the 70S bound to the antibiotics (PDB ID: 4V53 and 4WOI, respectively) [6,7]. Nucleotides A1492 and A1493 of the 16S rRNA along with A1912 of the 23S were also adapted from these structures after alignments. Similarly, coordinates of EVN and HygA were derived by aligning the 70S with the structures of EVN and HygA bound (PDB ID: 5KCS and 5DOY, respectively) [8,9].

The energy of each of the models was minimized using the steepest descent approach in GROMACS v4.5.4 with AMBERFF99S force fields [10-14]. Minimizations were performed in an explicit solvent system within TIP3P water molecules with 100mM NaCl with resolved Mg ions for 10 000 steps each. The energy of the A site tRNA, followed by water molecules and then the entire system, was minimized sequentially.

Structure-based model

Contacts between mRNA and aa-tRNA were scaled by 0.8 while contacts between aa-tRNA and the ribosome were scaled by 0.4, 0.6, or 0.8 to ensure base-pairing between the codon-anticodon occurs prior to reversable excursions of aa-tRNA [15,16]. As these simulations cannot resolve chemical reactions such as GTP hydrolysis this step of aa-tRNA selection was not included in the simulations. However, by employing a system where the non-bonded contacts between the mRNA and tRNA are stronger than the accommodated tRNA position we ensure that initial selection (formation of codon-anticodon base-pairs) occurs prior to tRNA accommodation. Therefore, a discrepant non-bonded contact strength set up allows us to mimic the gating between initial selection and accommodation provided by the GTP-hydrolysis step. During analysis of our simulations, we considered that GTP hydrolysis would separate initial selection from proofreading, therefore, only simulations where initial selection completed before accommodation were considered.

Although not utilized in the molecular simulations from this study, a user could introduce "non-native "contacts in the structure-based potential. "Non-native" contacts would be beneficial to stabilize an intermediate or a simulation endpoint. Introducing non-native contacts needs to be performed cautiously as inappropriate addition of non-native contacts could lead to artificially stabilized conformations and produce artifacts within the molecular simulations. The current study nonetheless reports on differences in the geometry of cognate and near-cognate tRNA selection pathways and the relative conformational sampling of these systems.

Time-scale estimations

Initial estimations of the structure-based simulation time-scales were derived from the diffusion of tRNA through the accommodation channel similar to Yang *et al*. 2019 [17]. The diffusion of tRNA was calculated by:

$$D(R) = \frac{1}{2} \lim_{\Delta t \to \infty} \frac{d}{dt} \langle \left(R_{elbow}(t) - R_{elbow}(t_0)\right)^2 \rangle \quad \text{(S1)}$$

The average denoted by ⟨ ⟩ was determined for every time interval where | R_{elbow} -R | < 0.5 A. Diffusion was determined for all 1 Å intervals of the R_{elbow} distance (27-56 Å) (Supplemental Fig. 10). The diffusion of tRNA was compared to the diffusion of tRNA in explicit solvent simulations from Yang *et al.* 2019 [17].

The estimated timescale of 1 ns was then compared with the simulation results to assess accuracy compared to biochemically derived rates of accommodation[15,18]. Accommodation frequency (Supplemental Table 1) was determined directly from the number of t_{ru} required for accommodation (A). The rate of accommodation from simulations was then estimated using the Arrhenius equation,

$$k = Ae^{\frac{-E_a}{RT}} \tag{S2}$$

Where k is the rate of accommodation and E_a is the previously determined activation energy[19].

SUPPLEMENTARY NOTES

Estimation of structure-based simulation timescales

To estimate the rate of aa-tRNA accommodation we compared the rate of diffusion of aa-tRNA from structure-based simulations and explicit solvent simulations in an approach similar to Yang *et al*. 2019 [17]. The average rate of diffusion for our structure-based simulations determined from equation S1 was ~0.1 $Å^2/\tau_{ru}$, where τ_{ru} is time in reduced units (Supplemental Fig. 10). The average diffusion rate from structure-based simulations was compared to the average diffusion rate of 1-20$\mu m^2/s$ of tRNA in the accommodation corridor estimated from explicit solvent simulations [17]. This would yield each τ_{ru} as 50 ps to ~1 ns. With a 1 ns/τ_{ru} timescale accommodation attempts of tRNA would yield an attempt frequency of ~0.1 - 2 μs^{-1}, similar to previously estimated values[19]. The frequency of accommodation attempts was measured as the number of frames required for the A-site tRNA CCA end to approach the P-site tRNA CCA end. With an accommodation frequency of 2 μs^{-1}, an estimated barrier of accommodation of 13 k_BT[19] and using the Arrhenius equation, this yields accommodation rates of ~5 s^{-1} similar to those biochemically determined[15,18]. Using these timescales, the frequency of conversion between CR to GA and GA to AC is estimated to be ~5 and 22 μs^{-1}, respectively. With previously determined rates for tRNA reversible motions between the CR to GA and GA to AC states (260 and 90 s^{-1}) we can equate the barrier to transition between states to be ~8 and ~7 k_BT with the Arrhenius equation, respectively[15].

EVN-induced stalled complexes reveal strategy for antibiotic development

Previous reports on EVN predicted that the antibiotic prevents accommodation by occluding the aa-tRNA accommodation corridor [8]. If this is the mechanism of EVN action then we would expect to have visualized a stalled aa-tRNA conformation unable to accommodate; however, we observed several intermediate conformations alongside accommodation of the aa-tRNA (Fig. 6 D). Our data show, for the first time, that the aa-tRNA stalls at different positions during accommodation as it tries to surpass the EVN barrier. It stalls at first contact, during compression, and through direct interactions with the groove of the tRNA at the elbow (Fig. 6 E). The binding of EVN to the groove of the tRNA is similar to the interaction of H89 with the tRNA during accommodation [16,20]. Our data supports a mechanism by which EVN is a steric

hindrance for accommodation and directly interacts with tRNA through the groove of the tRNA elbow. This finding indicates that EVN amplifies the canonical barrier of H89 to the accommodation of aa-tRNA. If amplifying canonical barriers is an effective strategy utilized by antibiotics, perhaps it is a strategy that can be employed for design of the next generation of antibiotics. For example, alongside the major groove that EVN interacts with, the adjacent minor groove of the tRNA is an available interaction surface where an antibiotic could interact with accommodating aa-tRNA. Development of an antibiotic to interact with the minor groove of the acceptor stem would likely inhibit or reduce the rate of aa-tRNA accommodation similar to EVN providing a target site for a novel antibiotic. Furthermore, we identified H71 as a barrier to near-cognate aa-tRNA, if this barrier were amplified similar to how EVN amplifies the H89 barrier, cognate aa-tRNA accommodation could be limited as near-cognate aa-tRNA is. Thus, these findings provide mechanistic strategies for development of novel antibiotics.

The ribosome treats cognate tRNA as near-cognate in the presence of HygA

The predicted mechanism of HygA is to prevent accommodation of the A-site aa-tRNA into the PTC. Our data supports this model, but also provides new insight into the mechanistic basis of how HygA is preventing accommodation. The main finding is that the positioning of the 3'-CCA end of the A site aa-tRNA is disrupted, either being too far from the PTC, bent back on itself, or directed towards the mRNA channel (Fig. 7A-C). Since the majority of simulations show that the 3'-CCA end is either folded back on itself or positioned away from the PTC, we suggest that the ribosome is treating the aa-tRNA as near-cognate. The average distance of the A-site and P-site A76 ($R_{cca-cca}$) is increased by 0.8Å in the presence of HygA for cognate tRNA (Supplemental Table 1). Although this is not quite the same distance of 9.6 Å for near-cognate, the observed $R_{cca-cca}$ distance is too far from the PTC to allow for peptide bond formation. Interestingly, HygA has little to no effect on the accommodation of near-cognate tRNA. The CCA ends adopt distances similar to that of near-cognate tRNAs without antibiotic at the end of the simulation (when convergence has occurred) (Supplemental Table 1). It is likely that the inherent large $R_{cca-cca}$ already observed for the near-cognate does not allow HygA to disrupt near-cognate accommodation.

Supplementary Table 1. Average number of time steps required for accommodation of the CCA end of A-site aa-tRNA and average distance between the A-site and P-site 3'-CCA ends ($R_{cca\text{-}cca}$).

	Accommodation frequency (μs)		$R_{cca\text{-}cca}$ distance, post-accommodation(Å)	
	Cognate	Near-cognate	Cognate	Near-cognate
No Antibiotic	2.4 ± 1.0 μs	2.3 ± 1.3 μs	6.3 ± 0.3	9.6 ± 0.4
GEN	1.3 ± 0.6 μs	1.1 ± 0.5 μs	6.9 ± 0.3	9.6 ± 0.4
NEO	1.3 ± 0.6 μs	1.8 ± 1.2 μs	6.9 ± 0.3	9.6 ± 0.3
EVN	1.7 ± 0.6 μs	1.3 ± 1.0 μs	6.9 ± 0.3	9.6 ± 0.4
HGR	2.8 ± 1.6 μs	1.4 ± 0.6 μs	7.1 ± 0.3	9.8 ± 0.4

Supplemental Table 2. $\theta_{t\text{-}m}$ values during aa-tRNA accommodation.

	Cognate $\theta_{t\text{-}m}$ (°)	Near-cognate $\theta_{t\text{-}m}$ (°)
No Antibiotic	132 ± 4	121 ± 1
		127 ± 2
		131 ± 3
EVN	87 ± 1	61 ± 5
	89 ± 2	100 ± 2
	100 ± 2	112 ± 2
	113 ± 1	118 ± 3
	117 ± 2	127 ± 2

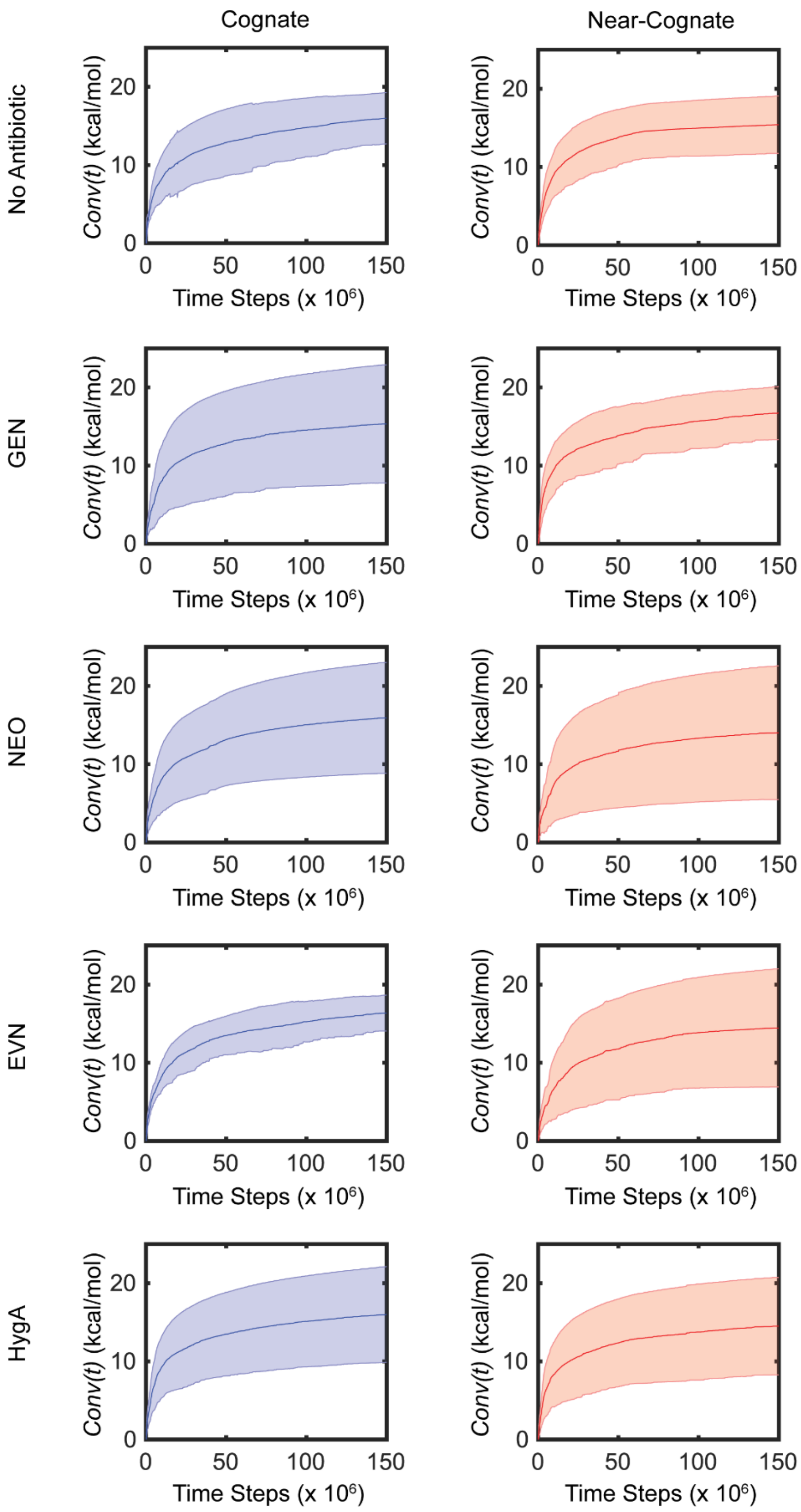

Supplementary Figure 1. Convergence of aa-tRNA accommodation simulations. Convergence of structure-based simulations was evaluated by the time dependent deviations of the system $\sigma(t)$. Convergence was considered when the $Conv(t)$ value approached a plateau value as describe in Vaiana *et al* [21]. Solid line is the average $Conv(t)$ value and the shaded area is the standard deviation of each simulations. Source data are provided as a Source Data file.

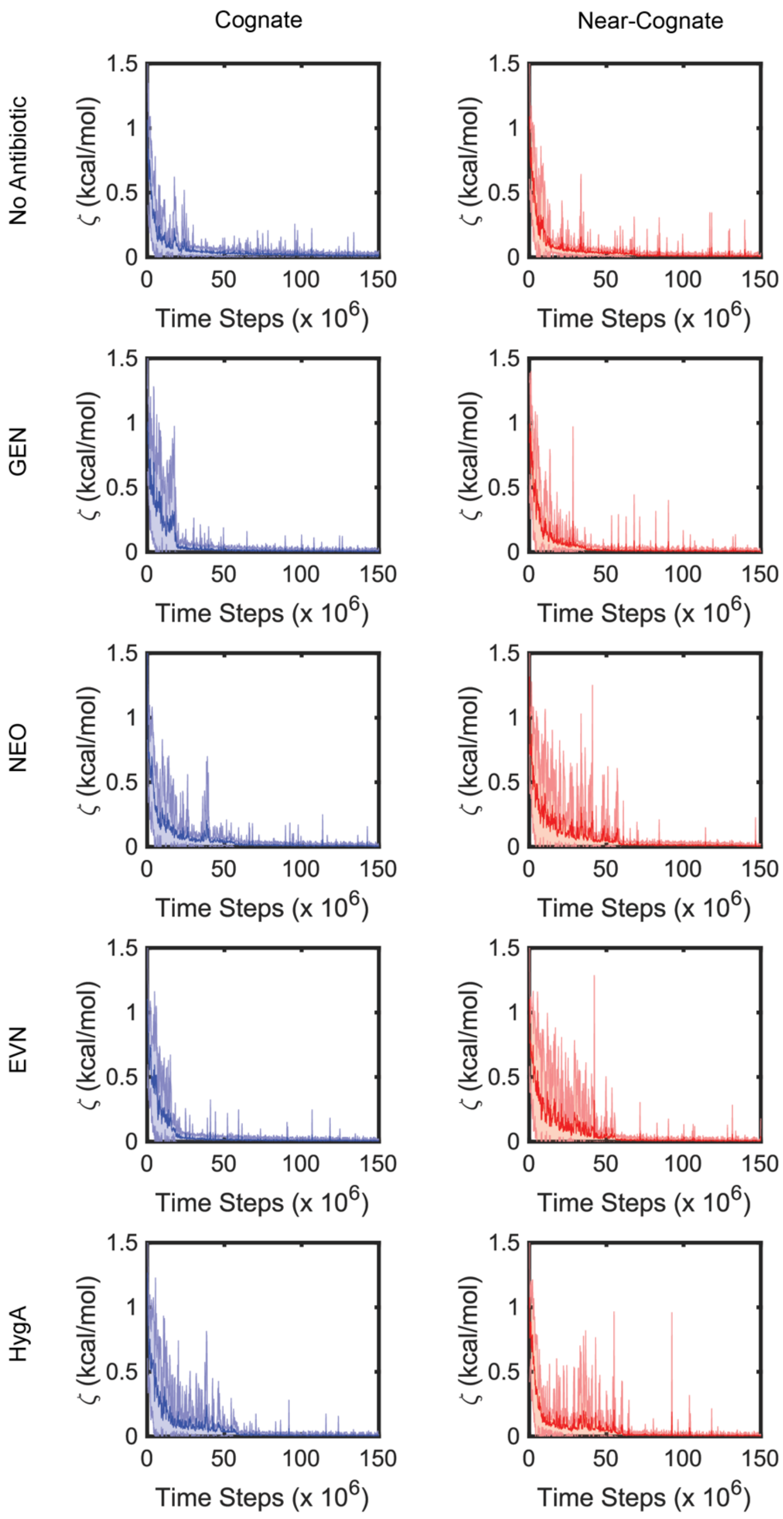

Supplementary Figure 2. Second measure of convergence of aa-tRNA accommodation simulations from average statistical fluctuation of free energy approximate landscapes. Convergence of structure-based simulations was evaluated with the statistical fluctuations of the free energy approximate landscapes $\zeta(t)$. Convergence was considered when the windowed time average value of $\zeta(t)$ decreased below 0.1[21]. Solid line is the average $Conv(t)$ value and the shaded area is the standard deviation of each simulations. Source data are provided as a Source Data file.

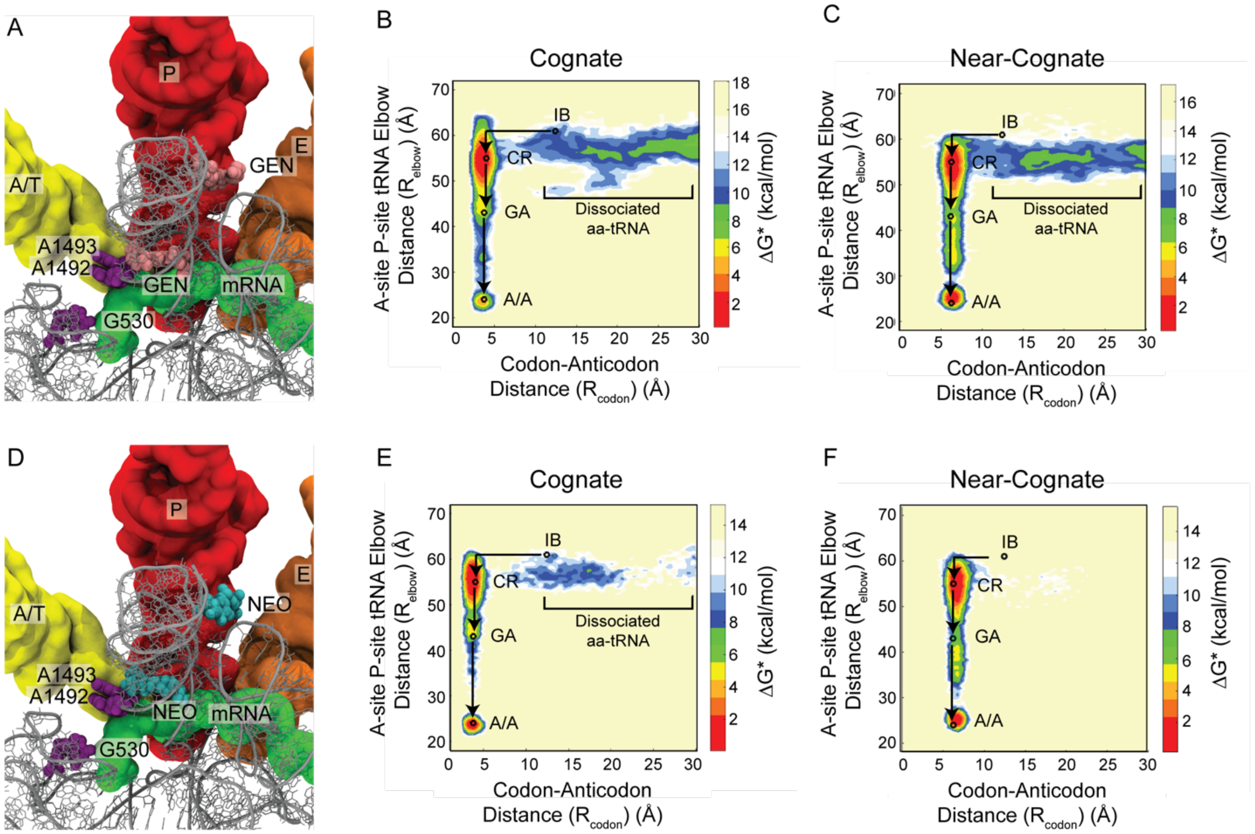

Supplementary Figure 3 Aminoglycosides preventing the alternative near-cognate aa-tRNA accommodation pathway. Visualization of (A) GEN and (D) NEO binding sites in h44 on the 16S rRNA and the extruded positions of A1492 and A1493. Boltzmann weighted free energy landscape approximations of accommodation defined by A site and P site tRNA elbow distances (R_{elbow}) and A site tRNA anticodon and mRNA codon distance (R_{codon}) for cognate in the presence of (B)-GEN, and (E)-NEO. Boltzmann weight free-energy landscape approximations of accommodation defined by R_{elbow} and R_{codon} for near-cognate accommodation in the presence of (C)-GEN and (F)-Neo. Source data are provided as a Source Data file.

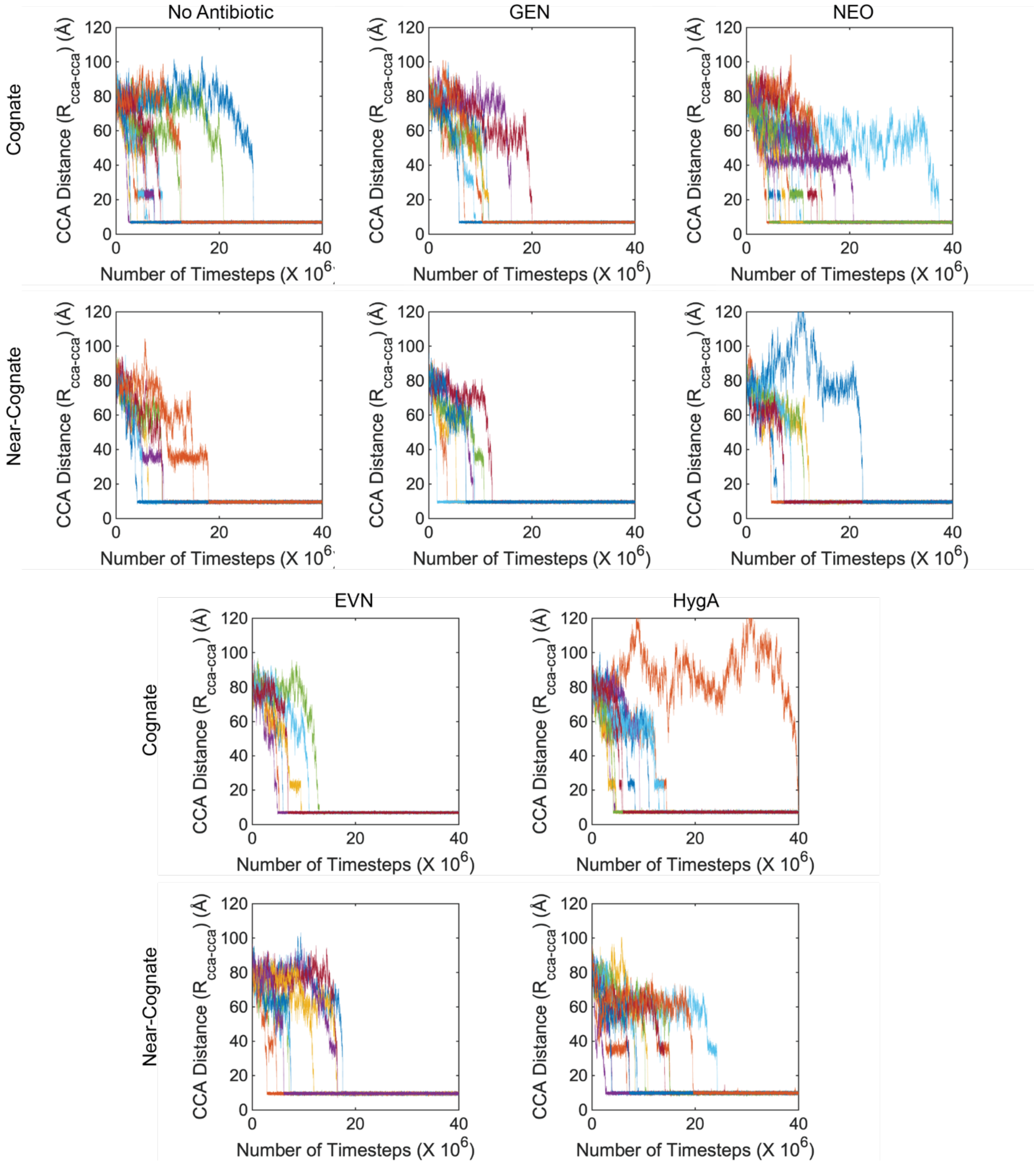

Supplementary Figure 4. Time dependance of $R_{cca\text{-}cca}$ during cognate and near-cognate accommodation in the presence and absence of antibiotics (100 simulations per case). $R_{cca\text{-}cca}$ measurements for each of the modelled systems used for simulation (presence or absence of antibiotic). Each graph shows 20/100 simulations for clarity purposes. The different colored traces represent unique simulations. Source data are provided as a Source Data file.

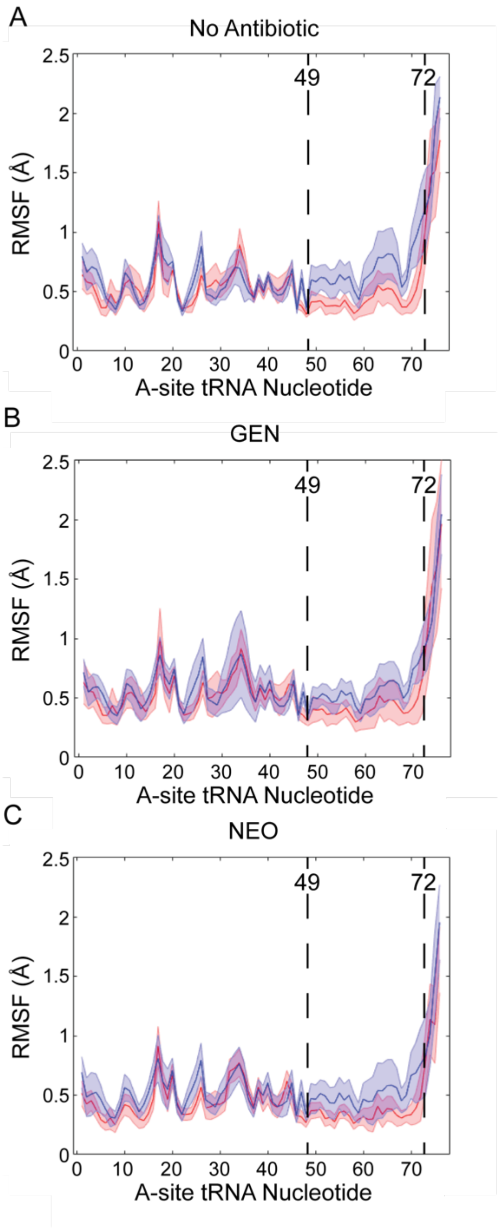

Supplementary Figure 5. Root means squared fluctuations (RMSF) of accommodated aa-tRNA in the presence of aminoglycosides. (A) RMSF of accommodated cognate (Red) and near-cognate (blue) aa-tRNA in the absence of antibiotics, nucleotides 49 to 72 highlighted. (B) RMSF of accommodated cognate (Red) and near-cognate (blue) aa-tRNA in the presence of GEN, nucleotides 49 to 72 highlighted. (C) RMSF of accommodated cognate (Red) and near-cognate (blue) aa-tRNA in the presence of NEO, nucleotides 49 to 72 highlighted. Solid line is the average RMSF value, and the shaded area is the standard deviation of each simulations. Source data are provided as a Source Data file.

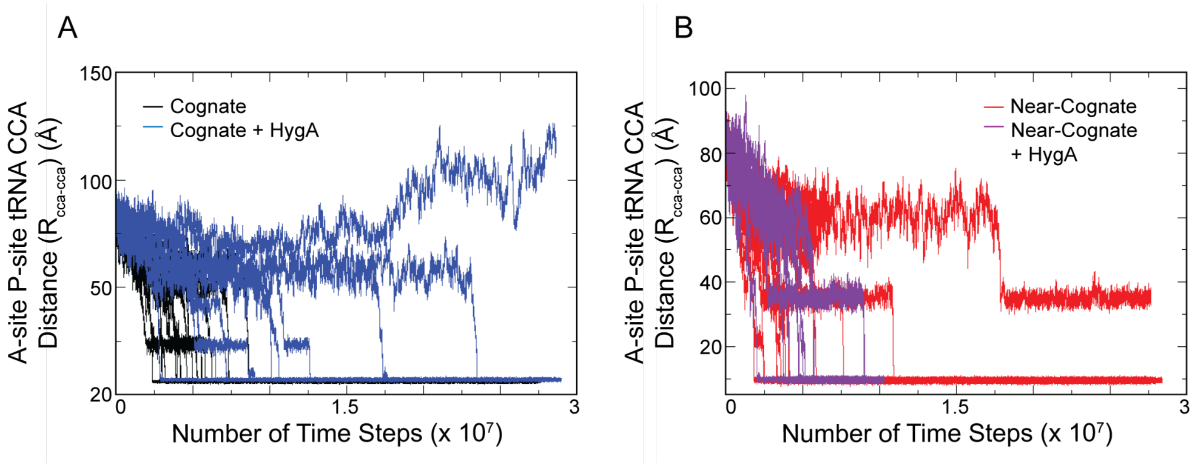

Supplementary Figure 6. Impact of HygA on the accommodation of cognate and near-cognate aa-tRNA. Measurement of aa-tRNA selection from simulations with HygA bound adjacent to the PTC, measured by the $R_{cca\text{-}cca}$ reaction coordinate for both (A) cognate and (B) near-cognate aa-tRNA in the presence and absence of HygA. Source data are provided as a Source Data file.

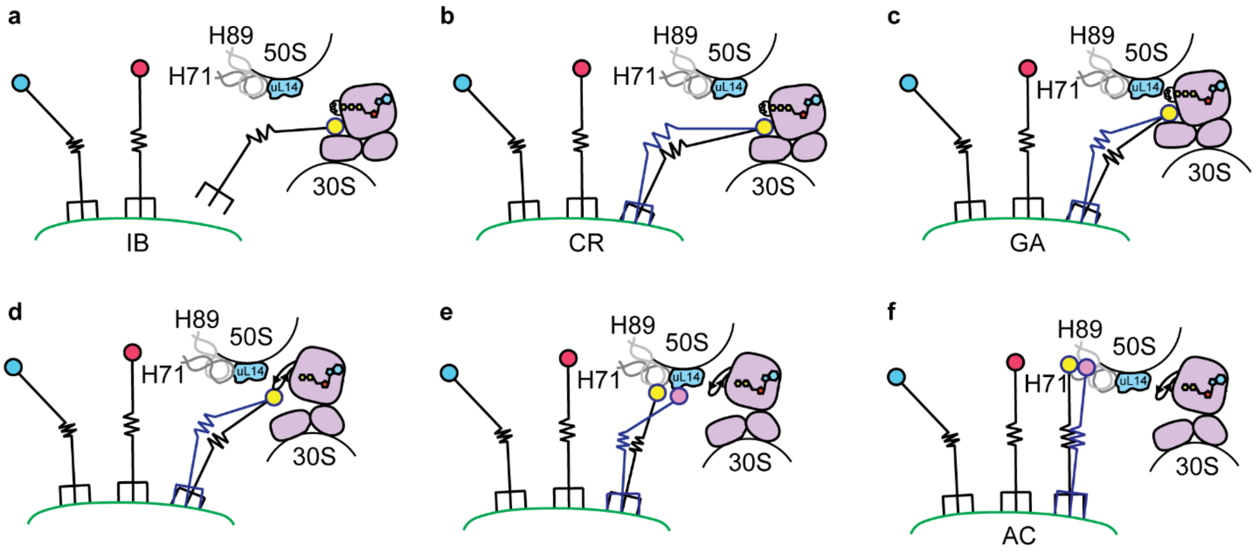

Supplementary Figure 7. Misalignment of near-cognate aa-tRNA during proofreading of tRNA selection. (a) EF-Tu (purple) delivers aa-tRNA to the ribosome by interacting with the 30S interface in the absence of codon-anticodon interactions between the mRNA (green) and aa-tRNA (cognate – black and yellow). (b) Codon-anticodon interactions are formed between the mRNA and aa-tRNA and at this point near-cognate aa-tRNA (blue) may become misaligned. (c) EF-Tu is docked onto the 50S of the ribosome (GA) and GTP hydrolysis is initiated. (d) aa-tRNA begins to release from EF-Tu, switch I is interacting with the acceptor stem of the aa-tRNA, and misalignment of near-cognate aa-tRNA (blue and pink) can already be detected. (e) Intermediates of aa-tRNA accommodation are observed where near-cognate aa-tRNA is misaligned interacting with uL14 and H71. (f) In the A site the near-cognate aa-tRNA is misaligned in comparison to cognate aa-tRNA.

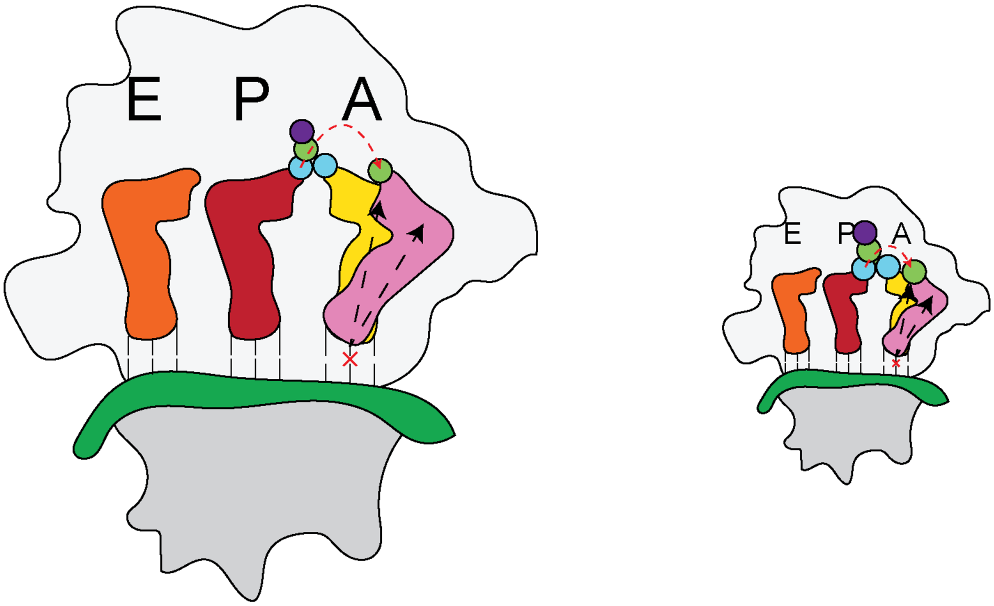

Supplementary Figure 8. Size requirements of the ribosome for aa-tRNA alignment/misalignment hypothesis. The effectiveness of a misaligned near-cognate aa-tRNA is dependent on the size of the tRNA and the ribosome. When ribosomes are relatively large on a molecular scale (Left) then a misalignment initiated from the codon-anticodon interactions between the mRNA (green) and aa-tRNA (yellow-cognate, pink-near-cognate) can be amplified through the length of the aa-tRNA (black arrows). This misalignment positions the amino acid (blue – cognate, green – near-cognate) such that a near-cognate amino acid is not properly positioned for peptide bond formation. In the context of a small ribosome (Right) the perturbance at the codon-anticodon interactions is not capable of resulting in a significant amino acid position change due to the relatively small length of the lever (tRNA).

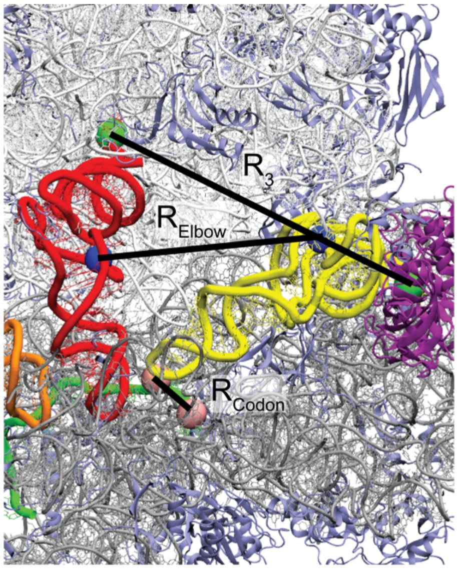

Supplementary Figure 9. Description of the distances used in calculations. Measurements for R_{Codon} (Distance between N1 of G in the wobble position on the mRNA and N3 of U34 in the tRNA), R_{Elbow} (Distance between O3' of U8 of the P-site tRNA and U60 of the A-site tRNA), R_3 (Distance between the center of mass of the A76 of the A-site and P-site tRNA).

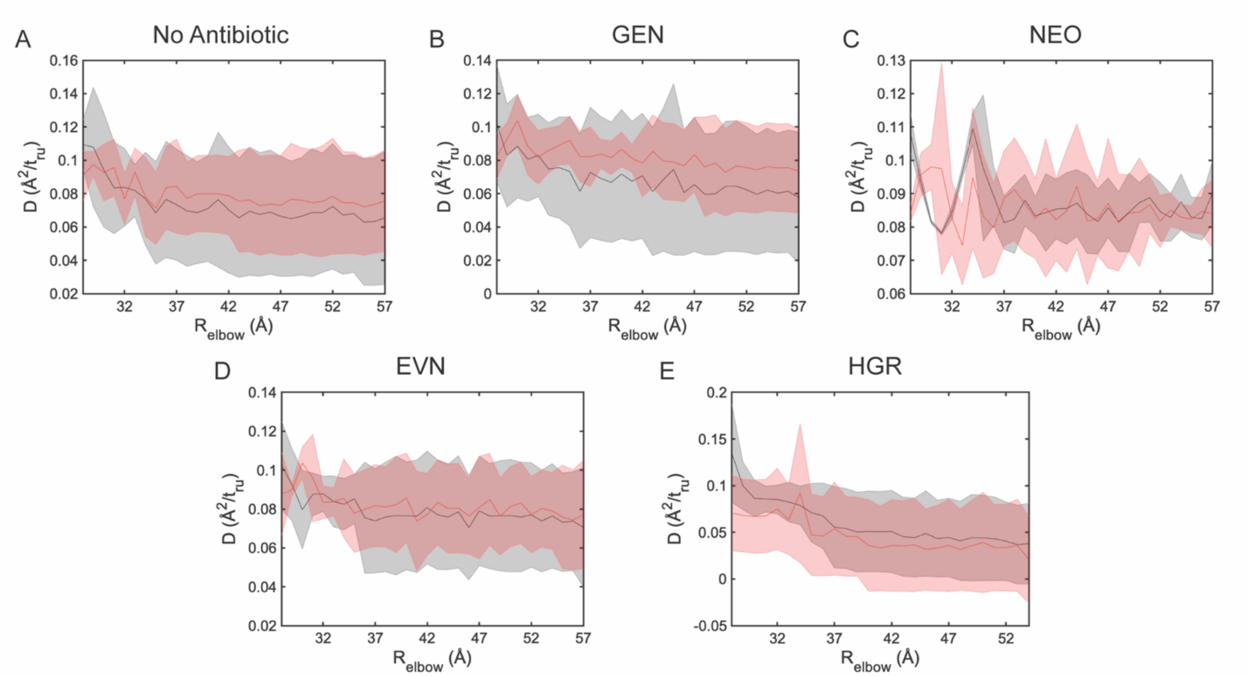

Supplementary Figure 10. Diffusions of A-site tRNA during accommodation. Diffusion of R_{elbow} calculated from eq 4 for 20 simulations of 100 million time-steps simulations for (A) no antibiotic, (B) GEN, (C) NEO, (D) EVN, (E) HGR, for both cognate (gray) and near-cognate (red) aa-tRNA. Average rates of diffusion were used to determine the time of each time-step in the structure-based simulation. Line indicates the average rate of diffusion and the shaded area is the standard deviation. Solid line is the average D value and the shaded area is the standard deviation of each simulations.

SUPPLEMENTARY REFERENCES

1 Loveland, A. B., Demo, G., Grigorieff, N. & Korostelev, A. A. Ensemble cryo-EM elucidates the mechanism of translation fidelity. *Nature* **546**, 113-117 (2017). https://doi.org:10.1038/nature22397

2 Song, H., Parsons, M. R., Rowsell, S., Leonard, G. & Phillips, S. E. V. Crystal structure of intact elongation factor EF-Tu from *Escherichia coli* in GDP conformation at 2.05Å Resolution. *J. Mol. Biol.* **285**, 1245-1256 (1999).

3 Kjeldgaard, M., Nissen, P., Thirup, S. & Nyborg, J. The crystal structure of elongation factor EF-Tu from *Thermus aquaticus* in the GTP conformation. *Structure* **1**, 35-50 (1993). https://doi.org:10.1016/0969-2126(93)90007-4

4 Humphrey, W., Dalke, A. & Schulten, K. VMD: visual molecular dynamics. *J Mol Graph Model* **14**, 33-38, 27-38 (1996). https://doi.org:Doi 10.1016/0263-7855(96)00018-5

5 Pettersen, E. F. *et al.* UCSF Chimera—A visualization system for exploratory research and analysis. *J. Comput. Chem.* **25**, 1605-1612 (2004).

6 Wasserman, M. R. *et al.* Chemically related 4,5-linked aminoglycoside antibiotics drive subunit rotation in opposite directions. *Nat. Commun.*, 1-12 (2015).

7 Borovinskaya, M. A. *et al.* Structural basis for aminoglycoside inhibition of bacterial ribosome recycling. *Nat. Struct. Mol. Biol.* **14**, 727-732 (2007).

8 Arenz, S. *et al.* Structures of the orthosomycin antibiotics avilamycin and evernimicin in complex with the bacterial 70S ribosome. *Proc. Natl. Acad. Sci. U S A* **113**, 7527-7532 (2016). https://doi.org:10.1073/pnas.1604790113

9 Polikanov, Y. S. *et al.* Distinct tRNA Accommodation Intermediates Observed on the Ribosome with the Antibiotics Hygromycin A and A201A. *Mol. Cell.* **58**, 832-844 (2015).

10 Hornak, V. *et al.* Comparison of multiple Amber force fields and development of improved protein backbone parameters. *Proteins* **65**, 712-725 (2006).

11 Wang, J., Cieplak, P. & Kollman, P. How well does a Restrained Eloectrostatic Potential (RESP) model perform in calculating conformational energies of organic and biological molecules? *J. Comput. Chem.* **21**, 1049-1074 (2000).

12 Van Der Spoel, D. *et al.* GROMACS: fast, flexible, and free. *J. Comput. Chem.* **26**, 1701-1718 (2005).

13 Hess, B., Kutzner, C., Van Der Spoel, D. & Lindahl, E. GROMACS 4: algorithms for highly efficient, load-balanced, and scalable molecular simulations. *J. Chem. Theory Comput.* **4**, 435-447 (2008).

14 Pronk, S. *et al.* GROMACS 4.5: a high-throughput and highly parallel open source molecular simulaton toolkit. *Bioinformatics* **29**, 845-854 (2013).

15 Geggier, P. *et al.* Conformational sampling of aminoacyl-tRNA during selection on the bacterial ribosome. *J Mol Biol* **399**, 576-595 (2010). https://doi.org:10.1016/j.jmb.2010.04.038

16 Whitford, P. C. *et al.* Accommodation of aminoacyl-tRNA into the ribosome involves reversible excursions along multiple pathways. *RNA* **16**, 1196-1204 (2010). https://doi.org:10.1261/rna.2035410

17 Yang, H. *et al.* Diffusion of tRNA insisde the ribosome is position-dependent. *J. Chem. Phys.* **151** (2019).

18 Gromadski, K. B. & Rodnina, M. V. Kinetic determinants of high-fidelity tRNA discrimination on the ribosome. *Mol Cell* **13**, 191-200 (2004). https://doi.org:10.1016/s1097-2765(04)00005-x

19 Whitford, P. C., Onuchic, J. N. & Sanbonmatsu, K. Y. Connecting energy landscapes with experimental rates for aminoacyl-tRNA accommodation in the ribosome. *J Am Chem Soc* **132**, 13170-13171 (2010). https://doi.org:10.1021/ja1061399

20 Noel, J. K. & Whitford, P. C. How EF-Tu can contribute to efficient proofreading of aa-tRNA by the ribosome. *Nat. Commun.* **7** (2016).

21 Vaiana, A. C. & Sanbonmatsu, K. Y. Stochastic gating and drug-ribosome interactions. *J. Mol. Biol.* **386**, 648-661 (2009).
