# Supplementary material for: Geometric alignment of aminoacyl-tRNA relative to catalytic centers of the ribosome underpins accurate mRNA decoding
Source: Nat Commun. 2023 Sep 11;14:5582. doi: 10.1038/s41467-023-40404-9 (PMC10495418; doi:10.1038/s41467-023-40404-9)
Supplement: Supplementary file 2 — Description of Additional Supplementary Files [file 41467_2023_40404_MOESM2_ESM.pdf]

Description of Additional Supplementary Files

File Name: Supplementary Movie 1
Description: tRNA selection of cognate aa-tRNA following the canonical pathway. Cognate aa-tRNA (blue) bound to EF-Tu (red) is delivered to the SSU of the ribosome (blue and purple) in the IB state in the absence of codon-anticodon base-pairs. The aa-tRNA forms codon-anticodon base-pairs with the mRNA (green) and the aa-tRNA and EF-Tu move towards the LSU (grey and green). aa-tRNA is released from EF-Tu and moves towards the PTC adjacent to the P-site tRNA (yellow).

File Name: Supplementary Movie 2
Description: tRNA selection of near-cognate aa-tRNA following the alternative non-canonical pathway. Near-cognate aa-tRNA (orange) bound to EF-Tu (red) is delivered to the SSU of the ribosome (blue and purple) in the IB state in the absence of codon-anticodon interactions. The aa-tRNA fails to form codon-anticodon base-pairs with the mRNA (green) before the aa-tRNA and EF-Tu move towards the LSU (grey and green). aa-tRNA is released from EF-Tu and moves towards the PTC adjacent to the P-site tRNA (yellow). While aa-tRNA is in the accommodation corridor the codon-anticodon base-pairs between the tRNA and mRNA form.

File Name: Supplementary Movie 3
Description: Non-productive tRNA selection of near-cognate aa-tRNA. Near-cognate aa-tRNA (orange) bound to EF-Tu (red) is delivered to the SSU of the ribosome (blue and purple) in the IB state in the absence of codon-anticodon interactions. The aa-tRNA fails to form codon-anticodon base-pairs with the mRNA (green) before the aa-tRNA and EF-Tu move towards the LSU (grey and green). aa-tRNA is released from EF-Tu and fails to move toward the PTC adjacent to the P-site tRNA (yellow).
